# Drug repurposing approach against chikungunya virus: an *in vitro* and *in silico* study

**DOI:** 10.3389/fcimb.2023.1132538

**Published:** 2023-04-27

**Authors:** Bhagyashri Kasabe, Gunwant Ahire, Poonam Patil, Madhura Punekar, Kusuma Sai Davuluri, Mahadeo Kakade, Kalichamy Alagarasu, Deepti Parashar, Sarah Cherian

**Affiliations:** ^1^ Bioinformatics Group, Indian Council of Medical Research (ICMR)-National Institute of Virology, Pune, Maharashtra, India; ^2^ Dengue & Chikungunya Group, Indian Council of Medical Research (ICMR)-National Institute of Virology, Pune, Maharashtra, India

**Keywords:** chikungunya virus (CHIKV), drug repurposing, structural and non-structural proteins, *in silico* screening, *in vitro* validation

## Abstract

The chikungunya virus (CHIKV) is an alphavirus transmitted by *Aedes mosquitoes*. There are no licenced antivirals or vaccines for treatment or prevention. Drug repurposing approach has emerged as a novel concept to find alternative uses of therapeutics to battle pathogens. In the present study, anti CHIKV activity of fourteen FDA-approved drugs was investigated by *in vitro* and *in silico* approaches. Focus-forming unit assay, immunofluorescence test, and quantitative RT-PCR assay were used to assess the *in vitro* inhibitory effect of these drugs against CHIKV in Vero CCL-81 cells. The findings showed that nine compounds, viz., temsirolimus, 2-fluoroadenine, doxorubicin, felbinac, emetine, lomibuvir, enalaprilat, metyrapone and resveratrol exhibit anti chikungunya activity. Furthermore, *in silico* molecular docking studies performed by targeting CHIKV structural and non-structural proteins revealed that these drugs can bind to structural protein targets such as envelope protein, and capsid, and non-structural proteins NSP2, NSP3 and NSP4 (RdRp). Findings from *in vitro* and *in silico* studies reveal that these drugs can suppress the infection and replication of CHIKV and further *in vivo* studies followed by clinical trials are warranted.

## 1 Introduction

Chikungunya virus (CHIKV), a single-stranded RNA virus belonging to the genus *Alphavirus*, family *Togaviridae*, causes chikungunya fever (CHIKF) and is transmitted by the bite of female *Aedes aegypti* and *Aedes albopictus* mosquitoes. It frequently occurs in tropical regions and affects millions of people worldwide (Suhrbier, 2019). CHIKF is characterised by high-grade fever, headache, polyarthralgia, nausea and vomiting, rashes, muscle pain, stiffness in joints and crippling joint pain (Silva et al., 2018; Amaral et al., 2020; Benjamanukul et al., 2021). There is no specific treatment or vaccine available to prevent this disease (da Cunha and Trinta, 2017). The only preventive method available is the control of vector mosquitoes. In some cases, CHIKF progresses to chronic debilitating polyarthralgia. The development of chronic debilitating polyarthralgia depends on multiple factors including viral load and a higher viral load is associated with persistent arthritis in mice models (Zhang et al., 2022). Reducing the viral load might prevent persistent arthritis and efforts are ongoing to design effective antiviral agents.

The symptoms of CHIKF also resemble that of dengue fever (DF), which is caused by dengue virus (DENV) and spread by the same vector mosquitoes. Since both CHIKF and DF share common symptoms, CHIKF is often diagnosed as DF. Since both diseases have identical symptoms during the initial phase of the disease, drugs targeting both viruses might provide an advantage in reducing the viral load thereby reducing disease severity. Our recent study identified FDA-approved drugs namely resveratrol, doxorubicin, lomibuvir, elvitegravir, and enalaprilat with anti DENV activity using systems biology approach and *in vitro* studies (Punekar et al., 2022). Since the drugs had anti-DENV activity, identifying anti-CHIKV activity would add more value to the drugs and might have utility in regions where both the viruses co-circulate and wherein co-infections are more prevalent. Hence, in the present study, the anti-CHIKV activity was investigated for resveratrol, doxorubicin, lomibuvir, elvitegravir, and enalaprilat. In addition, drugs that were identified on the basis of the dengue transcriptomics data set but without anti-dengue activity and few other drugs with reported anti-inflammatory/antiviral properties against other viruses, which were found non-cytotoxic to the cells, were also explored for their anti-CHIKV activity. In total, 14 FDA-approved drugs were thus investigated in this study. Furthermore, drugs with anti CHIKV activity were investigated for their *in-silico* interactions with CHIKV proteins to identify the possible mechanisms of the anti-CHIKV activity.

## 2 Materials and methods

### 2.1 Cells and virus

Vero CCL-81 (ATCC^®^ CCL 81™) cell line was used for the study and the cells were cultured in minimal essential medium (MEM, HiMedia^®^) with 10% foetal bovine serum (FBS, GibcoTM, New York, USA) and 1x antimycotic antibiotic solution (Sigma Aldrich^®^, St Louis, MO, USA) at 37°C and under 5% CO_2_. The CHIKV virus (Strain No.0615, 73), isolated in the Vero CCL-81 cell line, was employed in this study and 0.01 multiplicity of infection (MOI) was used for infection.

### 2.2 Stock preparation of compounds

Drug compounds were obtained commercially (Sigma Aldrich St. Louis, MO, USA) (**Table 1**). The stock solutions of the drugs (20 mM) were prepared using dimethyl sulfoxide and were stored at -20°C for further use.

**Table 1:** Pharmacological characteristics of the investigated FDA-approved drugs.

Sr. no.	Compound name	Pharmacological class	Reported activity
1	Temsirolimus	Antineoplastic	1. Antineoplastic (Bufalo et al., 2006; Bellmunt et al., 2008) 2. Antiviral- anti-SARS CoV-2 and anti-HBV (Saso et al., 2018; Mullen et al., 2021) 3. mTOR inhibitor (Malizzia and Hsu, 2008)
2	2-Fluoroadenine	Antineoplastic	1. Antineoplastic (Rai et al., 2000) 2. Immunosuppressant (Frank et al., 1999)
3	Doxorubicin	Antineoplastic	1. Antineoplastic (Tacar et al., 2012) 2. Antiviral (Johansson et al., 2006; De Palma et al., 2007; Coelmont et al., 2009; Kaptein et al., 2010.; Al-Motawa et al., 2020)
4	Felbinac	Anti-inflammatory	1. Anti-inflammatory (Bolten, 1991)
5	Metyrapone	Adrenal steroid synthesis inhibitor	1. 11β-hydroxylase enzyme inhibitor (Daniel et al., 2015) 2. Antidepressant (Jahn et al., 2004)
6	Enalaprilat	ACE inhibitor	1. Treatment of hypertension and hypertensive heart failure (Ayaz et al., 2016)
7	Emetine	Antineoplastic	1. Antineoplastic (Sun et al., 2019) 2. Antiviral (Andersen et al., 2020; Kumar et al., 2021)
8	Resveratrol	Anti-inflammatory	1. Antilipemic and antidiabetic (Szkudelska and Szkudelski, 2010) 2.Anti-inflammatory (Elmali et al., 2007; Das and Das, 2022) 3.Antineoplastic (Berman et al., 2017) 4. Alzheimer’s disease pathomechanism modulator (Anekonda, 2006) 5. Antioxidant and Anti-microbial (Filip et al., 2003) 6. Antiviral (Campagna and Rivas, 2010) 7. Cardioprotective (Bradamante et al., 2004)
9	Lomibuvir	Protease inhibitor	1. Anti-HCV activity (Abdurakhmanov et al., 2017; Li et al., 2018) 2. SARS-CoV-2 (Mohamed et al., 2021)

### 2.3 Cytotoxic effect of the compounds

The cytotoxic effects and CC50 values of nine drugs have been reported earlier (Punekar et al., 2022) and the drugs at the highest non-toxic concentrations were used for screening of anti-CHIKV activity. For the additional drugs, a cytotoxicity assay was performed using a 3-(4,5-dimethythiazol-2-yl)-2,5-diphenyl tetrazolium bromide (MTT) assay and the percent cell viability and CC50 values were calculated as described earlier (Punekar et al., 2022). A concentration range of 0.78 µM to 200 µM was used for each drug. CC50 values were calculated by non-linear regression analysis using GraphPad Prism software version 7 (Graph Pad Software Inc., San Diego, CA, USA).

### 2.4 Antiviral assay

The concentration at which the drugs showed ≥90% cell viability was used for evaluating the anti-CHIKV activity. The antiviral activity was assessed under different treatment settings (pre-treatment, co-treatment, and post-treatment) (Alagarasu et al., 2022). Briefly, for pre-treatment, the cells were incubated with various concentrations of drugs for 24 h and the drugs were removed by washing and the cells were infected. In co-treatment, the virus was incubated with the drugs for one hour and the virus drug mixture was used for infecting the cells. In case of post-treatment, the cells were infected first followed by treatment with the drugs. Under all conditions, the cells were incubated for 24 h after infection. At 24 h, the CHIKV strain used in this study exhibited maximum cytopathic effects as a result of viral infection and the virus multiplication entered the stationary phase at this point, and the viral count ultimately stops rising. Therefore, we set a time restriction of 24 h. A virus control (VC) in which the cells received no treatment was maintained for all treatment conditions. A MOI of 0.01, calculated based on the number of cells seeded per well, was used for infection. After incubation, the cells were freeze thawed to release the virus particles and the culture supernatant was clarified by centrifugation and stored at -80°C. Culture supernatants were used for assessing the titre of virus particles by focus forming unit (FFU) assay and RNA copy number by quantitative real-time RT-PCR (qRT-PCR). The antiviral assays were repeated for the compounds with anti CHIKV activity.

### 2.5 FFU assay and quantitative RT-PCR

FFU assay was used to the quantify the virus particles as described earlier (Panda et al., 2021; Patil et al., 2021). Approximately 35000 cells/well were seeded in a 96-well plate and incubated for 24 h to form a monolayer. Tenfold serial dilutions of the culture supernatants obtained from the antiviral assays were added to the monolayer and incubated for one hour. The cells were washed after incubation followed by addition of MEM with 2% FBS and 1.8% carboxymethyl cellulose and incubated at 37°C for 24 h in a CO_2_ incubator. The cells were washed with phosphate-buffered saline containing Tween 20 detergent (PBST) after incubation, and the cells were fixed with chilled acetone and methanol (1:1 ratio). Blocking was performed using a blocking buffer (1% bovine serum albumin dissolved in PBS) for 40 min at 37°C. Then the cells were washed again and incubated with diluted anti-CHIKV monoclonal antibody (MAb ClVE4/D9 clone) (Damle et al., 2016) (1:300 dilution) for 40 min, followed by the addition of anti-mouse IgG HRP conjugate (1:1000 dilution) and incubated for 40 min. The cells were washed again and the True Blue Peroxidase Substrate, (LGC Sercare, Milford, MA, USA) was added in dark and incubated for 15 min at room temperature. After the blue tinge formation, the substrate was removed, dried and the number of foci were counted to determine the virus titer.

The viral RNA copy number of CHIKV in the culture filtrate was quantified using a qRT-PCR assay as described earlier (Panda et al., 2021; Patil et al., 2021). A commercial viral RNA extraction kit (QIAmp Viral RNA mini kit, Qiagen, Hilden, Germany) was used for the extraction of viral RNA. The RNA was subjected to one-step qRT-PCR using a commercial kit (SuperScript III One-Step RT-PCR System with Platinum Taq DNA Polymerase; ThermoFisher Scientific, Waltham, MA, USA). Oligonucleotide sequences were used as reported earlier (Parashar et al., 2013). The qRT-PCR conditions included reverse transcription at 50°C for 30 min, followed by inactivation of reverse transcriptase and activation of Taq DNA polymerase at 95°C for 2 min, and 45 PCR cycles of 95°C for 15 s and 60°C for one minute. In addition to the viral RNA from culture filtrate, dilutions of *in vitro*-transcribed viral RNA with known copy numbers were also subjected to qRT-PCR for generating a standard graph using Ct values. The viral RNA copy numbers of culture filtrate from different culture conditions were calculated using the standard graph.

### 2.6 Time-of-addition assay

To find out the phase of virus life cycle at which the drug interferes, infected cells were treated with the effective drugs at different time points after infection (0, 3 and 6 hours). Briefly, monolayers of Vero CCL-81 cells grown in 24-well plate in MEM containing 2% FBS were infected with 0.01 MOI of CHIKV for one hour at 37°C with 5% CO_2_. After incubation, the cells were washed using 1x sterile PBS and directly treated with the drugs, followed by incubation for 24 h post infection at 37°C and 5% CO_2_. This is considered as 0 h time of treatment. For 3 h and 6 h, the cells were infected with CHIKV and incubated for one hour at 37°C for virus adsorption. After incubation, the cells were washed using 1x sterile PBS and incubated with MEM supplemented with 2% FBS for 3 h and 6 h respectively. After the respective incubation, the cells were again washed with PBS and were treated with the drugs, followed by incubation for 24 h post infection at 37°C and 5% CO_2_. After incubation, to find out whether the drugs affect virus release, the virus particles present in cell lysates (extracellular) and cells (intracellular) of the treated and control wells were collected separately after 24 h post infection and analyzed using FFU assay.

### 2.7 Immunofluorescence assay

To find out the influence of the drugs on the infectivity of cells by CHIKV, immunofluorescence assay was performed as described earlier (Tagore et al., 2022). Briefly, 2 × 10^5^ Vero CCL-81 cells per well seeded onto a coverslip placed in each well of a 24-well plate. After 24 h, the confluent monolayers formed were infected with 0.01 MOI of virus. Different concentrations of drugs were added to the wells post infection and incubated for 12 h at 37°C in a CO_2_ incubator. Then, the cells attached to the cover slips were fixed using methanol and acetone (1:1 ratio) for 20 minutes and blocked with 1% bovine serum albumin (BSA) followed by wash with PBST. The cells were then incubated with anti-CHIKV MAb (ClVE4/D9 clone) (in-house developed) (Damle et al., 2016) (1:50) followed by incubation with anti-mouse IgG secondary antibody conjugated with FITC (Sigma Catalog no. F0257-1ML, USA) (1:500) for 40 minutes. Then, the cover slips were mounted onto a drop of mowiol (mounting solution) with 4’, 6-diamidino-2-phenylindole, dihydrochloride, a nuclear stain, in the slides. The slides were examined under EVOS Floid cell imaging station with 20x magnification (Thermo Fisher Scientific, Bedford, MA, USA). ImageJ software was used to analyze the fluorescent images. In each coverslip, four to five randomly selected fields were used for counting infected and uninfected cells and the mean percent of infected cells were used for analysis.

### 2.8 Western blot

To investigate the expression of viral proteins, a western blotting assay was performed. Vero CCL-81 cells were infected with CHIKV and incubated for 24 hours. The cell cultures were subjected to different types of treatment with various concentrations of the appropriate drugs. Cells were lysed using the RIPA buffer and 2X laemmli buffer at 1:1 ratio. The cell culture lysates from uninfected, infected without treatment and infected treated cells were subjected to an electrophoretic run on 10% discontinuous SDS polyacrylamide gels followed by electroblotting onto nitrocellulose membranes. The membranes were then blocked for 30 minutes using phosphate-buffered saline (PBS) containing 5% bovine serum albumin. Following a wash, membranes were incubated overnight at 4°C with a 1:1000 diluted anti-CHIKV MAb (ClVE4/D9 clone). This monoclonal antibody is known to react with the capsid protein of CHIKV and was characterized and purified as described earlier (Damle et al., 2016). The membranes were thoroughly rinsed with tris-buffered saline with 0.1% Tween 20 (TBST) after overnight incubation (5 washes totaling a 20-minute soak with rocking), and then they were incubated with the appropriate horseradish peroxidase (HRP) conjugated anti-mouse IgG antibody for two hours while rocking. For developing the blot, ECL substrate (Thermo Scientific) was used in the dark. The image of the blot was captured using the Gel Documentation system (BioRad™, USA), and analysis was done. To quantify the protein signals (density) of the viral protein and housekeeping protein in each lane, ImageJ software was used. After calculating the signal values for each band, the background signals were subtracted from the signal. The relative level of viral protein was normalized to beta-actin (monoclonal antibody 15G5A11/E2, catalog no:MA1-140, Invitrogen, USA). Signal densities were determined using ImageJ software and were plotted using GraphPad Prism software version 7.

### 2.9 Viral attachment and entry assay

To find out the effect of drug (enalaprilat) which showed antiviral activity under cotreatment on virus attachment and entry, viral attachment and entry assays were performed using FFU assay. The foci were used to evaluate the effects of enalaprilat on attachment and entry, based on the fact that holding samples at 4°C can restrict the entry of the virus. For attachment assay, Vero CCL-81 cells were infected with CHIKV at a MOI of 0.01 at 4°C (which permits attachment but not entry) or at 37°C (which permits both attachment and entry) in the presence of enalaprilat at indicated concentrations (used for co-treatment) for 1 hour. The cells were then washed twice using 1x sterile PBS to remove the virus and drug. The infected cells were covered with 300 µl MEM supplemented with 2% FBS prior to incubation for 24 h.

For entry assay, Vero CCL-81 cells were first infected with CHIKV at 4°C for one hour and following incubation, the cells were washed with 1x sterile PBS (twice) and enalaprilat drug at different concentrations was added on to the cells, the cells were then incubated at 37°C for two hours with 5% CO_2_. After two hours, the cells were again washed using PBS and the cells were covered with 300 µl MEM supplemented with 2% FBS prior to incubation for 24 h.

For both assays, after incubation, the freeze thawed clarified culture filtrate was used to find out the virus titre employing FFU assay.

### 2.10 Statistical analysis

The virus titre in terms of FFU, viral RNA copy number, percent infectivity and band area density were compared between virus control (cultures which did not receive treatment) and cultures treated with drugs using one-way ANOVA with correction for multiple comparisons. A p value less than 0.05 was considered significant. All analysis was performed using GraphPad Prism software version 7 (Graph Pad Software Inc., San Diego, CA, USA).

### 2.11 Homology modelling and structure validation of CHIKV targets

The two-dimensional structures of all the drug compounds were retrieved from PubChem (Kim et al., 2016). All available crystal structures of the CHIKV targets were downloaded from the Protein Data Bank (PDB) (Berman et al., 2000). These included the CHIKV capsid protein (5H23.PDB), the CHIKV envelope glycoprotein complex in its mature form (3N42.PDB) (Voss et al., 2010), the CHIKV NSP3 macrodomain bound with ADP-ribose (3GPO.PDB) (Malet et al., 2009), the CHIKV peptidase C9 domain of NSP2 (3TRK.PDB) and the CHIKV NSP2 helicase domain (6JIM.PDB) (Law et al., 2019). SWISS-Model was used for homology modelling of the CHIKV NSP1v-methyaltransferase and NSP4 RdRp domain (Waterhouse et al., 2018) (https://swissmodel.expasy.org/). The structural modelling of proteins was performed using the protein sequences of the CHIKV targets obtained from the NCBI GenBank (accession no. ARB19731) (Clark et al., 2016). The PDB Basic Local Alignment Search Tool (BLAST) was used to locate the appropriate templates for protein modelling.

The PDB files of the generated models were submitted to the respective servers for the structural analyses. The modelled protein structures were further validated using ProSA (Protein Structure Analysis; https://prosa.services.came.sbg.ac.at/prosa.php) (Wiederstein and Sippl, 2007) and quality assessment of the models were done. In SWISS model structure assessment, the structures are further analyzed using Ramachandran plot analysis and the Molprobity score. The Z-score was determined by the ProSA web tool to measure the deviation of the total energy of the structure with respect to an energy distribution derived from random conformations found in native proteins and available at the PDB. Using the Qualitative Model Energy Analysis (QMEAN), the stability of the modelled 3D structures were assessed (Khare et al., 2022).

### 2.12 Molecular docking

#### 2.12.1 Receptor and ligand preparation

For *in silico* studies, all the CHIKV targets were processed using the Schrodinger drug discovery suite (Version2022, -2). 3-D chemical structures of nine compounds were downloaded from Pubchem (Kim et al., 2016) and imported in Maestro (Version 13.3). The compounds were prepared using the LigPrep module of the Schrodinger suite. The default parameters were applied (force field, OPLS4; pH, 7.20.2). The prepared compounds were used in the viral screening protocol. 3D structures of targets were downloaded from the RCSB PDB. The protein preparation wizard (Friesner et al., 2004) was used to refine and energy-minimize the proteins after importing the protein crystal structures into Maestro 13.5 to make sure they were structurally correct. The SiteMap (Halgren, 2009) module in Schrodinger contains all possible pocket identifications of all protein structures. Those SiteMap pockets showing a drugability score (D-score) of >0.8 were selected for docking and grid generation (Randhawa et al., 2022). Residues from site map analysis were used to generate grids for docking. The receptor grid generation module was used to generate a grid box surrounded by SiteMap-selected residues by keeping the default parameters of Vander Waals forces scaling factor 0.08 and charge cut-off 0.15, subjected to an OPLS3 force field (Lewis, 2004; Nair et al., 2012).

#### 2.12.2 Virtual screening workflow

The Glide virtual screening workflow was used to search perfect fit of the compound to CHIKV targets that interact and properly suit the active site of the target protein. The Schrödinger modules were used for High Throughput Virtual Screening (HTVS) on the Schrödinger small molecule database. Schrödinger’s Glide module includes a procedure for virtual screening and all docking calculations. It makes use of a grid-based ligand docking technique and provides a number of compound poses/positions as output. Energy-minimized positions were used for the final scoring, and the compounds with the best docked poses and the largest negative score values were chosen for future investigations (Vetrivel et al., 2021).

#### 2.12.3 MM-GBSA and ligand efficiency calculations

Using the molecular mechanics generalized born surface region (MM-GBSA) module in Schrodinger involving nine compounds and seven targets, the binding free energy (Gbind) of the docked complexes was calculated. (Schrodinger Suite, LLC, 2017-4; New York, NY, USA). Simultaneously, rotamer search methods, the VSGB solvent model, and the OPLS2005, force field were used to estimate the binding free energy and the ligand efficiency (ln values) using the equations as provided earlier (Teng et al., 2021, Sharma et al., 2022).

## 3 Results

### 3.1 Effect of shortlisted repurposed drugs on the proliferation of Vero CCL-81 cells

MTT assay was performed to test the cytotoxicity of 16 FDA-approved drugs. A total of 14 drugs showed ≥90% cell viability at their maximum non-toxic concentration while two drugs i.e. Low-dose naltrexone (LDN) and Masitinib were found to be highly toxic even at their lowest concentrations and were not processed further. Maximum nontoxic dose of each drug was selected for primary screening (**Table 2**). The effects of different drugs on cell viability with CC50 values are provided in **Table 2** and **Figure S1**.

**Table 2:** CC50 values and maximum non-toxic concentration of the drugs.

S. No.	Drug name	CC50 value ± SE (µM)	The maximum nontoxic concentration used for primary screening of drugs against CHIKV (µM)
1	NU-1025	NA	100
2	Temsirolimus	413.4 ± 378.5	50
3	Doxorubicin	116.9 ± 8.246	25
4	2-Fluroadenine	436.7 ± 320.4	100
5	Emetine	NA	200
6	Metyrapone	5599 ± 6096	200
7	Felbinac	525 ± 200.2	3.125
8	Lomibuvir	31.48 ± 9.627	6.25
9	Enalaprilat	83.04 ± 18.71	1.56
10	3- isobutyl 1- methylxanthine	177.2 ± 21.67	50
11	Lenalidomide	NA	200
12	5-α-3-β- androstanol	23.51 ± 0.856	12.5
13	Elvitegravir	12.72 ± 1.515	6.25
14	Resveratrol	40.86 ± 4.566	12.5

NA, Not Applicable.

### 3.2 Primary screening of repurposed drugs for anti CHIKV activity

A total of 14 drugs were assessed for anti-CHIKV activity in primary screening under pre, co and posttreatment conditions. Virus output after treatment was measured using FFU assay. The results revealed that nine drugs showed inhibitory activity against CHIKV.

**Figure 1:**
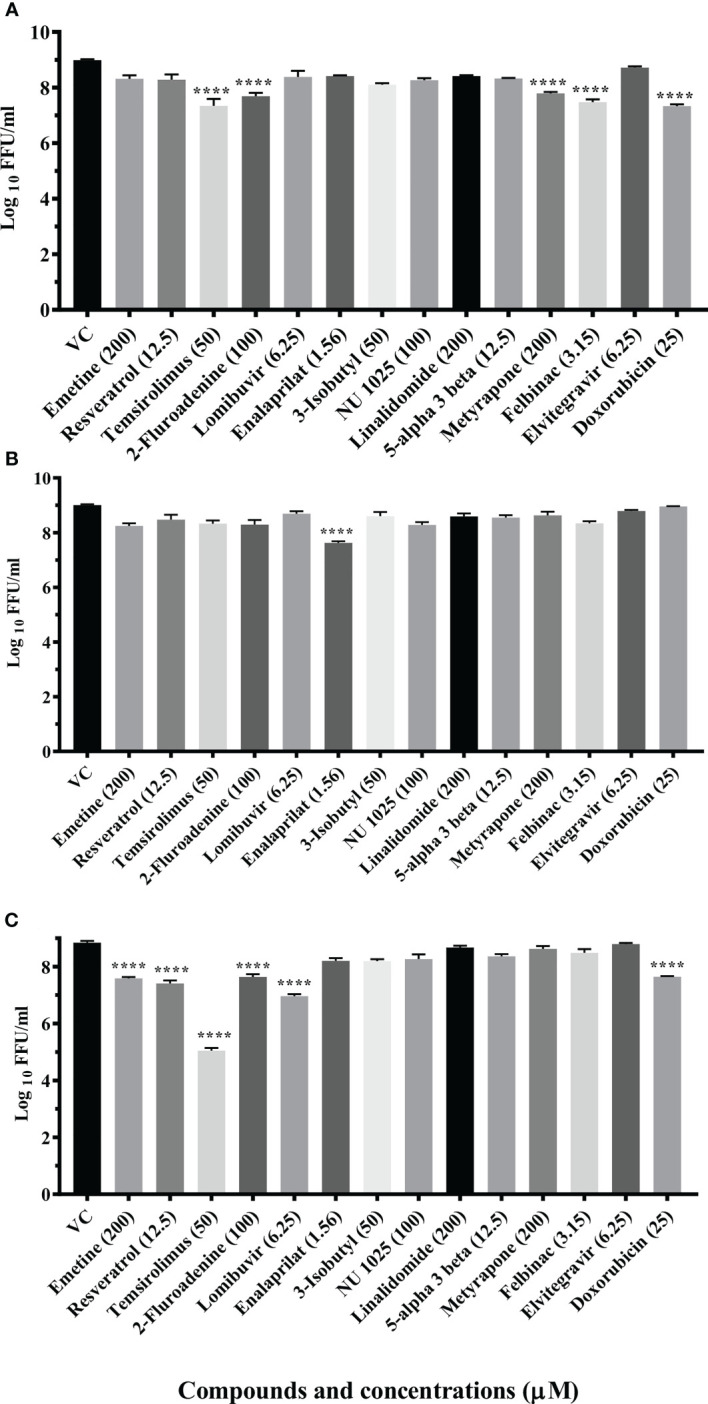
Primary screening of repurposed drugs at highest nontoxic concentration against CHIKV pretreatment **(A)**, cotreatment **(B)** and posttreatment **(C)** conditions. Vero CCL-81 cells treated with drugs under pre, co and posttreatment conditions at the highest maximum nontoxic dose and incubated for 24 (h) Plates were frozen after incubation, and culture filtrates were utilized for the FFU assay. The trials were carried out in triplicate at two different time points, and the outcomes were expressed as mean log_10_ focus-forming unit/ml ± standard error. All the treatment conditions were compared with the VC. ****p < 0.0001.

Three drugs (temsirolimus, 2-fluoroadenine and doxorubicin) were found to be effective under pre and posttreatment conditions. Two drugs (felbinac and metyrapone) were found to be effective in pretreatment conditions. Three drugs (emetine, resveratrol and lomibuvir) were found to be effective in only posttreatment condition. Enalaprilat was found to be effective in cotreatment condition (**Figure 1**).

### 3.3 Effect of different concentrations of repurposed drugs against CHIKV under pre and posttreatment conditions and evaluation of time-of-addition under posttreatment condition

The drugs which showed anti-CHIKV activity under pre and posttreatment conditions were further assessed at different concentrations to find out the concentration of the drug which exerts 50% inhibition (IC50) of CHIKV. The selectivity index and IC50 values for each drug under different conditions have been provided in **Table 3** and **Figure S2**.

**Table 3:** Summary of effective inhibition under different treatment conditions.

Sr. no.	Drug name	CC50 (µM)	Maximum concentration (µM)	Log difference effectiveness against CHIKV	IC50 (µM)	Selectivity Index (SI)
1	Temsirolimus	413.4	50	Pretreatment- 2.316 Posttreatment- 5.192	4.857 6.043	85.11 68.41
			25	Pretreatment-1.386 Posttreatment- 0.967	4.857 6.043	85.11 68.41
2	2-fluoroadenine	436.7	100	Pretreatment- 1.48 Posttreatment- 1.184	9.586 7.592	45.56 5.340
			50	Pretreatment- 1.381 Posttreatment- 1.187		
3	Doxorubicin	116.9	25	Pretreatment- 1.332 Posttreatment-1.494	6.896 6.408	16.95 18.24
			12.5	Pretreatment- 1.205 Posttreatment- 1.361		
			6.25	Posttreatment- 1.112		
4	Felbinac	525	3.125	Pretreatment- 1.625	0.238	2205.88
			1.56	Pretreatment- 1.49		
5	Metyrapone	5599	200	Pretreatment- 1.55	12.23	457.80
			100	Pretreatment- 1.529		
6	Enalaprilat	83.04	1.56	Cotreatment- 1.142	0.496	167.42
			0.78	Cotreatment- 0.897		
7	Emetine	>200	200	Posttreatment- 1.564	4.237	47.2
			100	Posttreatment-1.32	4.237	47.2
			50	Posttreatment-1.068	4.237	47.2
8	Resveratrol	40.86	12.5	Posttreatment- 1.384	1.721	23.74
			6.25	Posttreatment- 0.609	1.721	
9	Lomibuvir	31.48	6.25	Posttreatment- 1.215	1.587	19.83

#### 3.3.1 Temsirolimus

A significant reduction in the mean log_10_ viral titer/ml from 8.665 in VC to 6 and 7.279 was observed in cells pretreated with 50 μM and 25 μM concentrations of temsirolimus respectively (p< 0.0001). A significant 0.809 log_10_ titer (p< 0.0001) decrease in viral RNA copy number was observed at 50 µM concentration compared to VC (**Figure S3A**)

**Figure 2:**
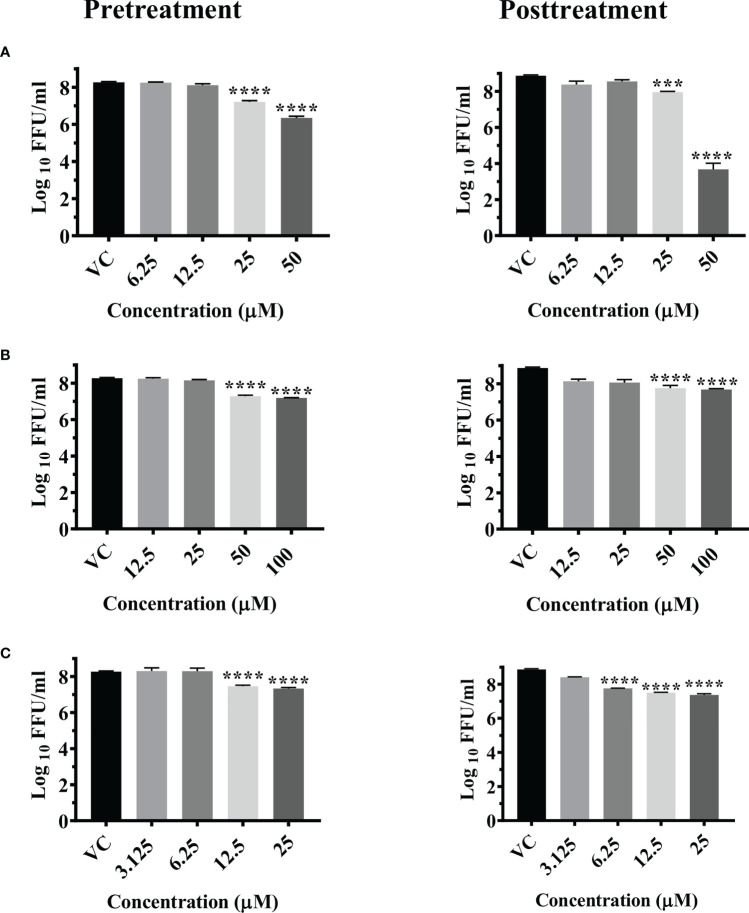
Antiviral effect of temsirolimus **(A)**, 2-fluoroadenine **(B)** and doxorubicin **(C)** against CHIKV under pre and posttreatment conditions. Vero CCL-81 cells were pre and post treated with different concentrations of drug and incubated for 24 hrs. After incubation, culture supernatants were used for the FFU assay. The trials were carried out in triplicate at two different time points, and the outcomes are expressed as mean log_10_ focus forming unit/ml ± standard error. All the treatment conditions were compared with the VC. **** (p<0.0001), ***(p<0.001).

Under posttreatment conditions, significant reduction in the mean log_10_ viral titre/ml from 8.870 in VC to 3.678 and 7.903 was observed in cells treated with 50 µM and 25 µM concentrations of the drug respectively (**Figure 2A**). A significant 2.487 log_10_ titer decrease in RNA copy number of CHIKV was observed at 50 µM concentration compared to VC (**Figure S3A**).

Time of addition experiments revealed that temsirolimus was effective at all the three time points (0, 3 and 6 h) tested (**Figures S4A**–**S6A**). At 0 h and 3 h, 50 µM temsirolimus exerted significant reductions in extracellular (~4 log_10_) and intracellular (~3 log_10_) virus titers compared to the respective VC groups. At 6 h, the 50 µM concentration showed significant reductions in extracellular (~ 3 log_10_) and intracellular (~2 log_10_) virus titers compared to the respective VC groups. Inhibition of both intra- and extracellular viral titers suggest that the drug does not act at the virus release step.

#### 3.3.2 2-Fluoroadenine

Significant reductions in the mean log_10_ viral titer/ml from 8.665 in VC to 7.185 and 7.284 were observed in cells pretreated with 100 µM and 50 µM concentrations of 2-fluoroadenine respectively (p< 0.0001) (**Figure 2B**). Pretreatment with 100 µM 2-fluoroadenine resulted in a significant reduction of viral RNA copy number compared to VC (p< 0.0001) (**Figure S3B**).

In case of posttreatment, significant reductions in the mean log_10_ viral titer/ml from 8.870 in VC to 7.686 and 7.0 were observed in cell cultures treated with 50 µM and 100 µM drug concentrations respectively (**Figure 2B**). Time of addition assay revealed that this drug was effective at all three time points (0, 3, 6 h) (S4b, S5b, S6b). In cultures that received 100 µM 2-Flouroadenine, ~1 log10 reduction in intra- and extracellular virus titer was observed compared to respective cultures that received no treatment. Mild reduction in intra- and extracellular virus titers was also observed in infected cells treated with 25 and 50 µM drug concentrations compared to the respective VC groups.

#### 3.3.3 Doxorubicin

The pretreatment of cells with 25 and 12.5 µM concentrations of doxorubicin led to significant reductions in the mean log_10_ viral titer/ml from 8.665 in VC to 7.333 and 7.460 respectively (**Figure 2C**).

Posttreatment of cells with doxorubicin resulted in a significant reduction in the mean log_10_ viral titer/ml from 8.870 in VC to 7.376, 7.509 and 7.758 at 25 µM, 12.5 µM and 6.25 µM concentrations compared to the VC respectively. (**Figure 2C**). There was no decrease in the viral RNA copy number in both the treatment conditions (**Figure S3C**).

At a concentration of 25 µM, doxorubicin exerted reduction in both intra- and extracellular virus particles at different time points of addition (0, 3 and 6 h) after infection. Under 12.5 and 6.25 µM concentrations of the drug, the reduction in intracellular virus titer was not as prominent as extracellular virus titer compared to respective VC. With increasing time points of drug addition, the reduction in viral titer lessened (**Figures S4C**–**S6C**).

### 3.4 Effect of different concentrations of repurposed drugs against CHIKV under pre and cotreatment conditions

#### 3.4.1 Felbinac

**Figure 3:**
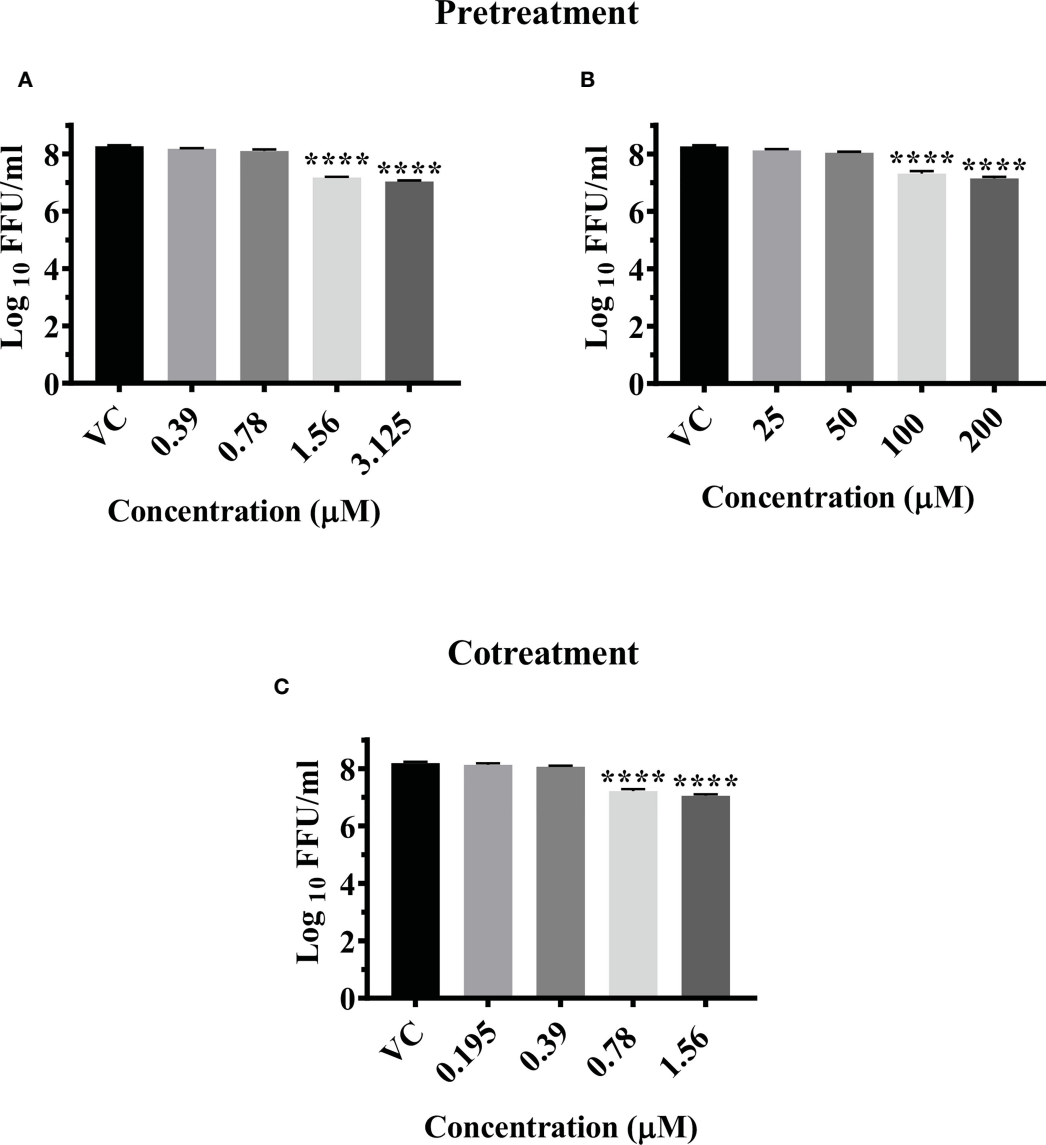
Antiviral effect of felbinac **(A)**, metyrapone **(B)** under pretreatment condition and Enalaprilat **(C)** under cotreatment condition against CHIKV. Vero CCL-81 cells were pre and co treated with different concentrations of drug and incubated for 24 hrs. After incubation. culture supernatants were processed for the FFU assay. The trials were carried out in triplicate at two different time points, and the outcomes are expressed as mean log10 focus forming unit/ml ± standard error. All the treatment conditions were compared with the VC. **** (p<0.0001).

Pretreatment of cells with 3.125 µM and 1.56 µM concentration of felbinac reduced the mean log_10_ virus titre/ml from 8.665 in VC to 7.040 and 7.175 respectively (P<0.0001) (**Figure 3A**). The drug did not have any effect on viral RNA titre (**Figure S3D**).

#### 3.4.2 Metyrapone

Pretreatment of cells with 200 µM and 100 µM concentrations of metyrapone significantly reduced the mean log_10_ FFU/ml virus titre to 7.115 and 7.136 compared to the VC titre (8.665) (p<0.0001) (**Figure 3B**). The log_10_ viral RNA copy number reduced from 9.670 in VC to 8.984 in cells pretreated with 200 µM metyrapone (p<0.0001) (**Figure S3E**).

#### 3.4.3 Enalaprilat

Cotreatment of cells and virus with 1.56 µM and 0.78 µM concentrations of enalaprilat exerted significant reduction in the mean log_10_ FFU/ml viral titer from 8.194 in VC to 7.052 and 7.297 respectively (p< 0.0001) (**Figure 3C**). No significant reduction was observed for viral RNA titer (**Figure S3F**).

**Figure 4:**
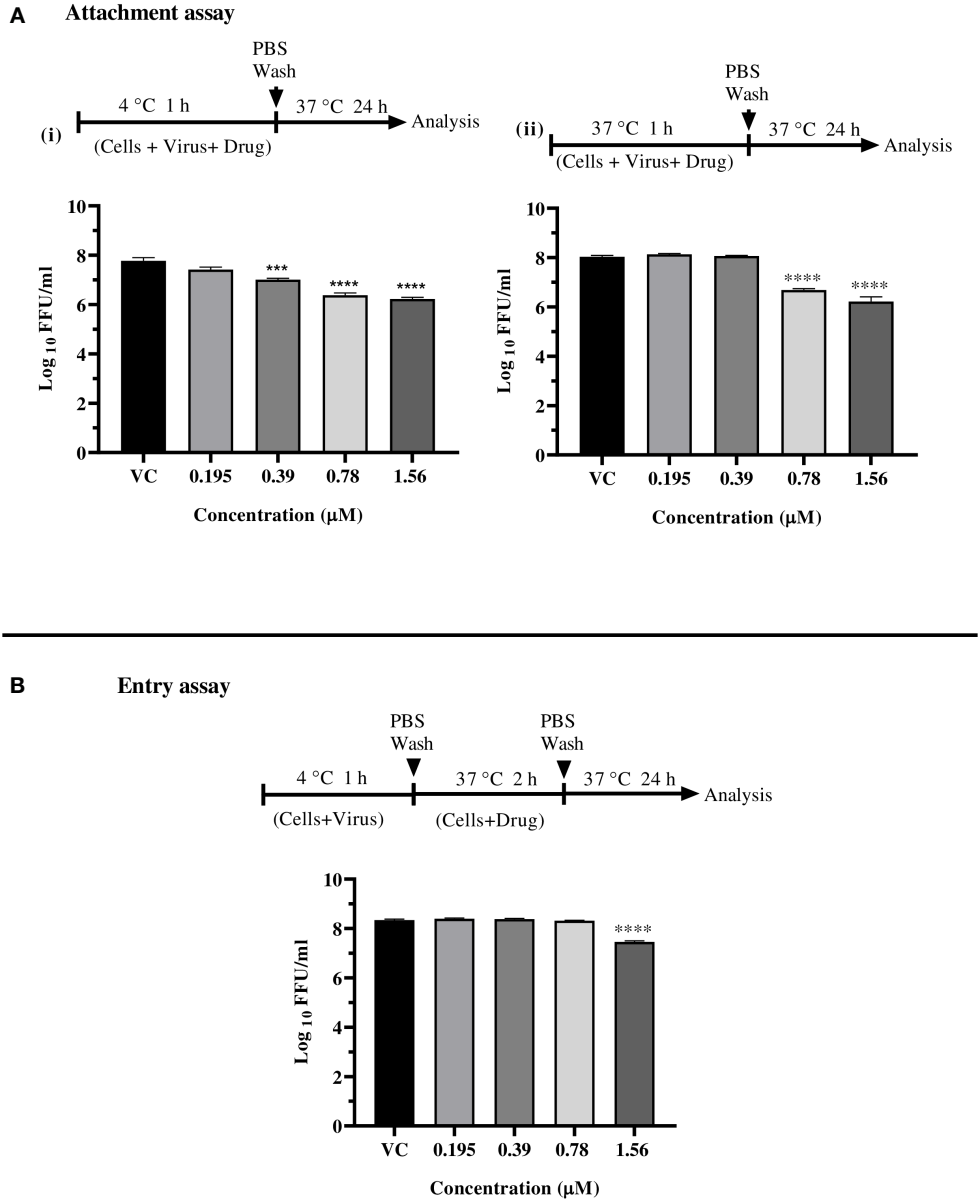
Effect of enalaprilat against virus attachment and entry. **(A)** Attachment assay at 4°C (i) and 37°C (ii)- The experimental procedure, virus concentration, and the time of addition and treatment with the drug are presented for CHIKV in schematics and the graphs. The log_10_ values were determined by counting the number of foci and were compared with the virus control group. Each data point is the mean log_10_ focus forming unit/ml ± standard error. ****p< 0.0001. **(B)** Entry assay at 4°C - The log_10_ values were determined by counting the number of foci and compared to that of the virus control group. Each data represents the mean log_10_ focus forming unit/ml ± standard error. ***p<0.005, **** p<0.0001.

Since enalaprilat exerted anti-CHIKV activity under the cotreatment condition, further experiments were conducted to explore the effect of the drug on virus attachment to cell and virus entry. At concentrations of 1.56 µM and 0.78 µM, enalaprilat was shown to influence virus attachment at both 4^°^C and 37^°^C. (**Figure 4A** i, and ii). The virus entry assay showed that the drug affects the virus titer at 1.56 µM concentration (**Figure 4B**). These observations suggest that the drug affects virus attachment rather than virus entry. The reduction observed at the higher concentration in virus entry assay could be due to the effect of enalaprilat on virus particles that were not attached to the cells during 4°C incubation.

### 3.5 Effect of different concentrations of repurposed drugs against CHIKV under posttreatment condition

#### 3.5.1 Emetine

**Figure 5:**
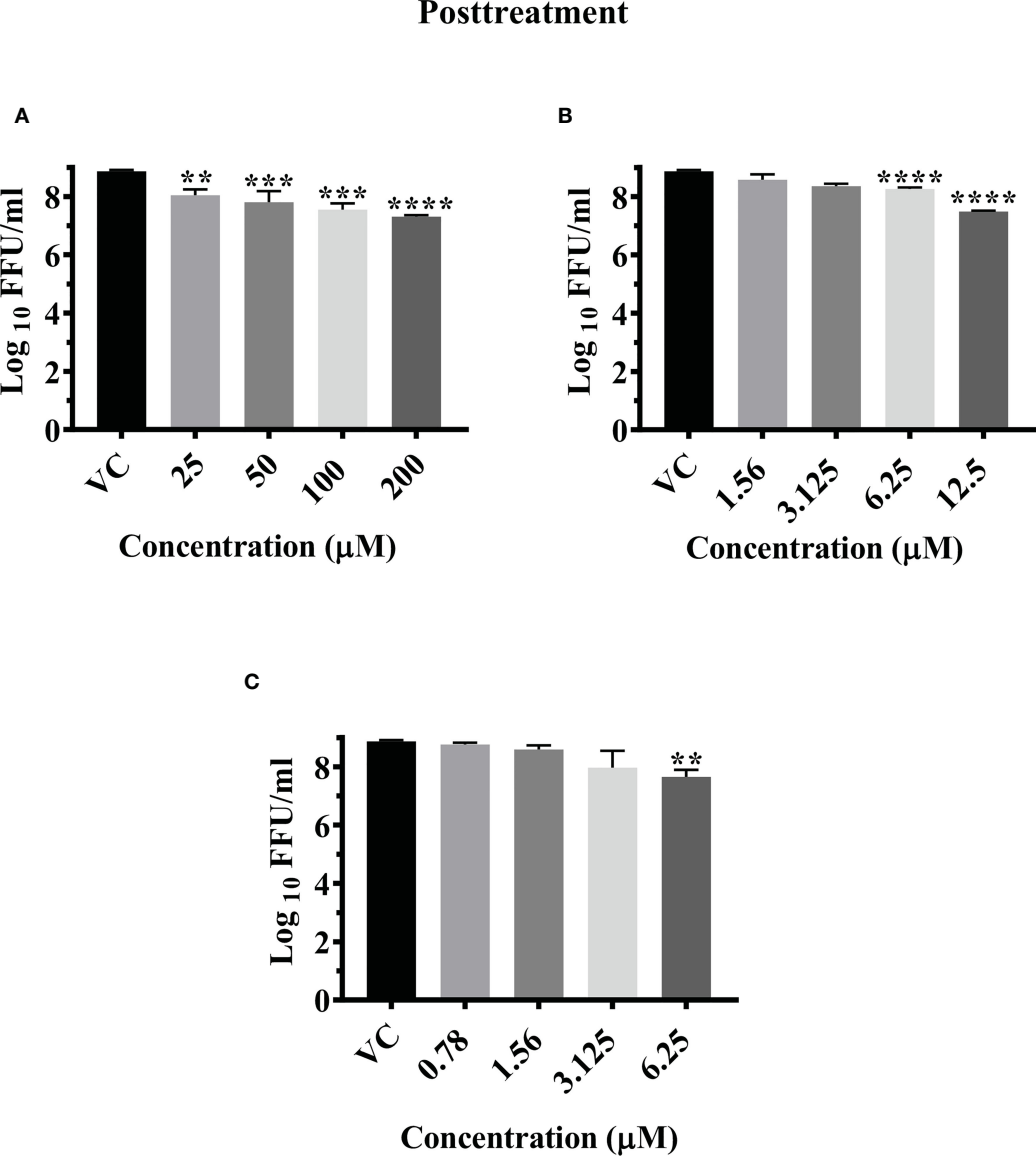
Antiviral effect of emetine **(A)**, resveratrol **(B)** and lomibuvir **(C)** against CHIKV under posttreatment condition using IFA. Infected Vero CCL-81 cells were treated with different concentrations of drug post infection and incubated for 24 hours and plates were frozen and culture supernatants were used for the FFU assay (The trials were carried out in triplicate at two different time points, and the outcomes are expressed as mean log_10_ foci forming unit/ml ± standard error). All the treatment conditions were compared with the VC. **** (p<0.0001), ***(p<0.001), **(p<0.01).

Post infection treatment of cells with emetine significantly decreased the mean log_10_ FFU/ml viral titer from 8.870 in VC to 7.306, 7.550, and 7.802 at 200 µM, 100 µM and 50 µM concentrations respectively (p< 0.0001) (**Figure 5A**). No significant reduction in viral RNA titer was observed in cells treated with the drug compared to the VC (**Figure S3G**).

Time of addition experiments unveiled that emetine significantly reduced both intra- and extracellular virus titer when added at 0 and 3 h time points post infection at concentrations ranging from 50-200 µM compared to respective VC. Addition of emetine at 6 h time point led to significant reduction of intra- and extracellular virus titre at 200 µM concentration compared to the respective VC (S4d, S5d and S6d).

#### 3.5.2 Resveratrol

Post infection treatment of cells with resveratrol significantly lowered the mean log_10_ viral titer/ml from 8.870 in VC to 7.486 and 8.261 at 12.5 µM and 6.25 µM concentrations respectively (p<0.0001) (**Figure 5B**). No reduction in viral RNA titer was observed in cells treated with resveratrol (**Figure S3H**).

Addition of 12.5 µM resveratrol at 0, 3 and 6 h time points post infection resulted in significant decrease in both intra- and extracellular virus titer compared to the respective VC (S4e, S5e and S6e). At 6.25µM concentration, a mild but significant reduction in extracellular titer of virus was observed at 3 and 6 h time points.

#### 3.5.3 Lomibuvir

Post infection treatment of cells with 6.25 µM lomibuvir decreased mean log_10_ viral titer/ml from 8.870 in VC to 7.655 (P<0.001) (**Figure 5C**). There was no change observed in the titer of viral RNA when cells were treated with lomibuvir (**Figure S3I**).

Time of addition experiments unveiled that 6.25 µM lomibuvir significantly reduced both intra- and extracellular virus titer when added at 0, 3, and 6 h time points post infection compared to the respective VC. A small but significant reduction in virus titer was also observed in infected cultures treated with 3.125 µM concentration of the drug compared to the respective VC at 3 and 6 h time points (S4e, S5e and S6e).

### 3.6 Effect of different drugs on CHIKV infection in Vero CCL-Cells under posttreatment condition

**Figure 6:**
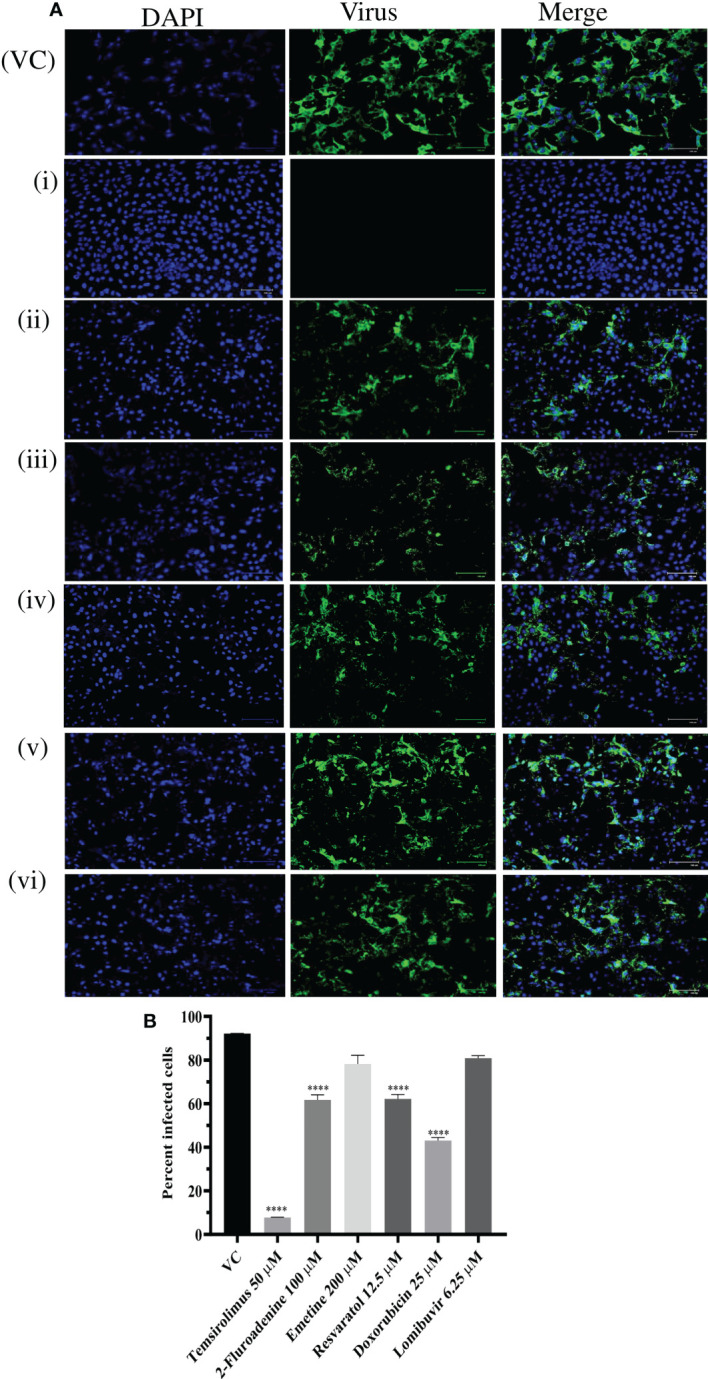
Effect of different drugs i.e. Temsirolimus (i), 2-fluoroadenine (ii), emetine (iii) resveratol (iv) doxorubicin (v), lomibuvir (vi) on CHIKV infection under posttreatment condition using IFA. Images represent CHIKV infected Vero CCL-81 cell lines under posttreatment condition. Cells infected with virus appear green in colour **(A)** Percentage of infected Vero CCL-81 cells in cultures treated with different concentrations of drug under posttreatment condition **(B)** All the treatment conditions were compared with the virus control. ****p < 0.0001. VC, virus control; CC, cell control: DC, drug control.

Since six drugs exerted anti-CHIKV activity post infection with higher reduction in virus titer, the results were further validated by IFA analysis. IFA results showed that these drugs significantly reduced the percent of infected cells compared to VC (**Figures 6A, B**). Reduction in percentage of infected cells was prominently observed in cells treated with temsirolimus, 2-fluoroadenine, resveratrol, and doxorubicin drugs.

### 3.7 Effect of different drugs on viral protein synthesis in CHIKV infected cells under posttreatment conditions

**Figure 7:**
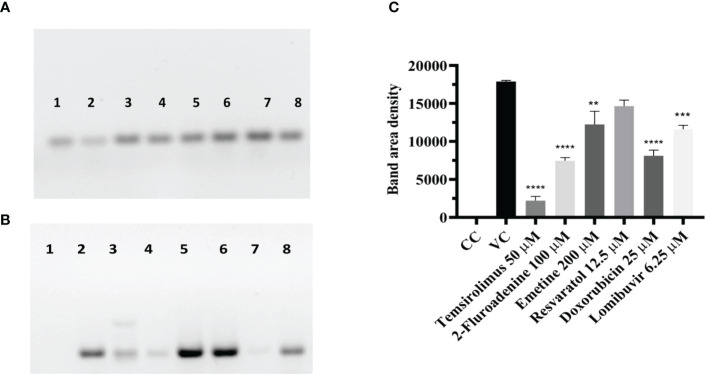
Effect of different drugs on CHIKV infection under posttreatment condition using western blot **(A)** β-actin expression in the cell lysates. **(B)** Antigens probed with the anti-CHIKV MAb. The MAb specifically detected the C protein of CHIKV expression which was normalized using the reference loading control β-actin, western blot image comparing the uninfected cells, infected cells (posttreatment with different drugs **(C)**. Bar plot of the intensities of capsid determined *via* ImageJ analysis from supernatants of treated and untreated cells (mean + SD, n=3 replicates). CHIKV antigen probed with a cell lysate of CHIKV-infected Vero cells shows the high-density band in lane-2. 2-Fluroadenine, temsirolimus and doxorubicin showed a significant reduction of C protein levels **p<0.01, ***p<0.001, ****p<0.0001 respectively (Lane-2,3,7). CHIKV antigen probed with lysate from emetine, resveratrol and lomibuvir, treated cells showing bands of 30-35kDa corresponding to the C protein of CHIKV (Lane-4,5,6,8) respectively. CHIKV antigen probed with cell control (uninfected cells), showing no reaction (Lane-1).

The lysates from CHIKV infected cell cultures which were treated with different drugs at their maximal non-toxic concentration post infection were subjected to western blot analysis. Following SDS-PAGE, cell lysates from CHIKV infected, uninfected, and drug treated cells were probed with the anti-CHIKV MAb. The MAb specifically detected the C protein of CHIKV. Though viral protein levels were reduced in cells treated with different drugs, the cells treated with 2-fluroademine and temsirolimus showed a significant reduction in the viral antigen expression compared to VC (**Figures 7A–C**).

### 3.8 Validation and evaluation of the CHIKV modelled protein structures

**Figure 8:**
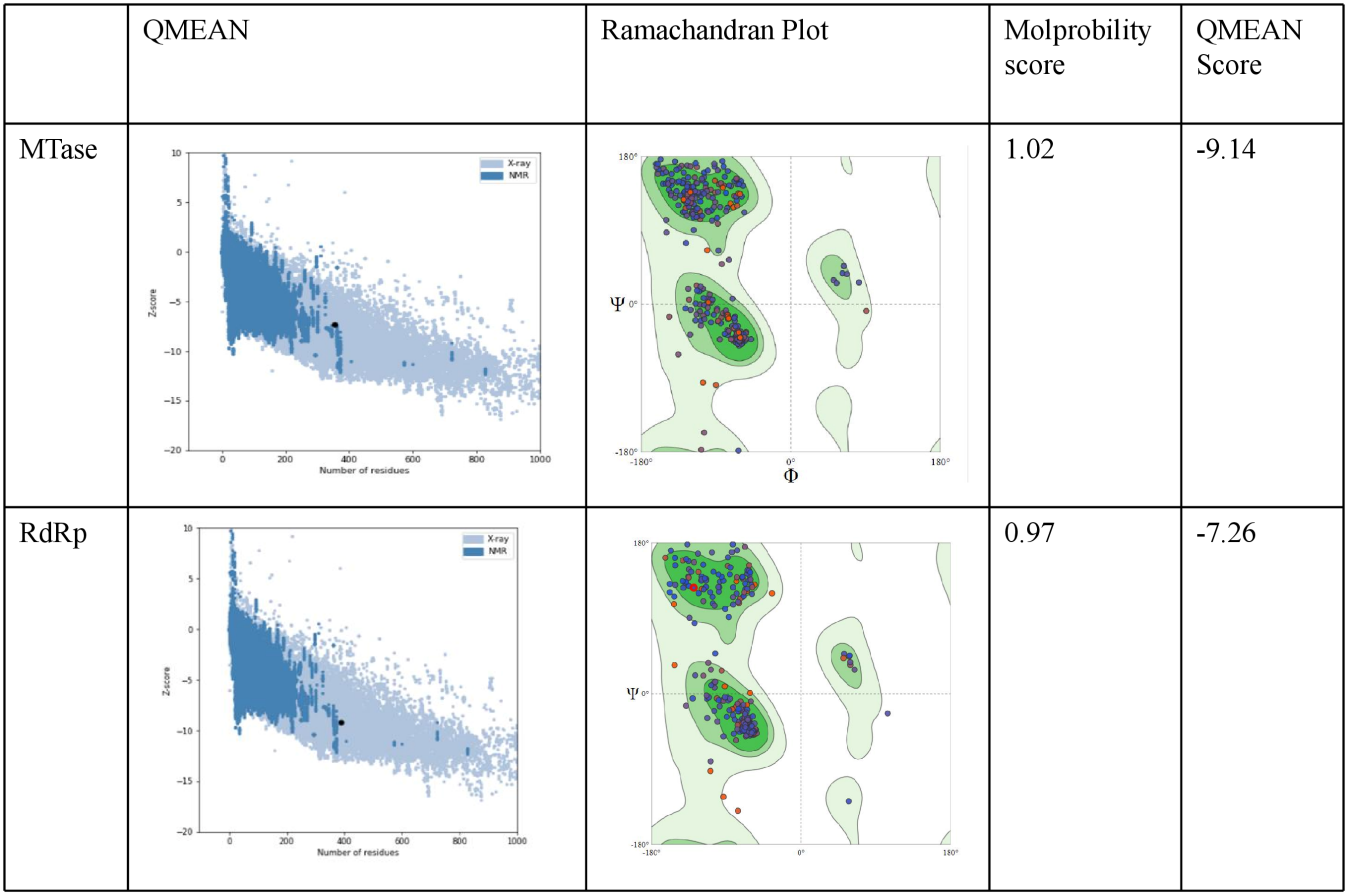
Evaluation of the modelled protein structures of CHIKV Methyl transferase (MTase) and RdRp using ProSA. As shown in the figure the Z-score was-9.14 for the predicted model of NSP1 Methyltransferase domain. Ramachandran plot of NSP1 Methyltransferase domain displays most residues in the favoured regions (c). The Z - score was -7.26 for the predicted model of RdRp. Ramachandran plot analysis of NSP2 helicase domain indicates residues in the favoured, allowed and generously allowed regions with90% of residues falling into the most favoured region.

The ProSA analysis of the three modelled CHIKV proteins displays that the Z-score values of the obtained models are located within the space of proteins determined by X ray and NMR structures. This suggests that the obtained models are reliable and close to experimentally determined structures. The results of the structure assessment through SWISS-Modelling analysis was observed in the form of Ramachandran plot, Molprobity score and QMEAN score for the two modelled proteins. The MTase modelled structure shows relatively few residues have phi/psi angles that fall within the disallowed region. The percentage of residues in the allowed region was observed to be 93.79%. The z score from Prosa web server shows -7.26 which represents an overall good quality of the model. The Ramachandran plot for the modelled RdRp displays 96.12% residues in the favored region and 0.78% residues as outliers. The Prosa z-score of -9.14 indicates a best quality model (**Figure 8**).

### 3.9 Molecular docking analysis of repurposed drugs with CHIKV target proteins

The nine compounds among the 14 identified drugs, which showed inhibitory activity against CHIKV in cell cultures under different treatment conditions, were subjected to molecular docking against the non-structural proteins of CHIKV viz. NSP1-NSP4 and structural proteins viz. mature envelope glycoprotein (EP) which includes E1, E2 and the capsid protein (**Table 3**). The most widely used technique for determining binding affinity and structural stability is to calculate binding free energy using the MM/GBSA method. The MM/GBSA technique used in the Prime Module of Schrodinger was used to determine the binding free energy of the various docked complexes. From MM/GBSA computations, a total of 18 conformations that covered the stable trajectories of the molecular dynamics (MD) production were taken. **Table 4** and **Table 5** displays the various energy components that were determined through the computation.

**Table 4:** Molecular docking interactions of the nine FDA approved drugs with CHIKV structural and non-structural proteins based on the binding affinity values and best pose. 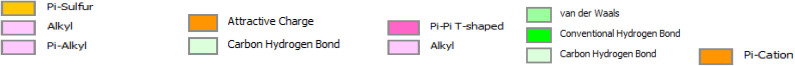

Compound	Potential binding viral targets		Docking score	Binding energy (kcal/mol)	Ligand Efficiency (kcal/ mol)
2-Fluroadenine	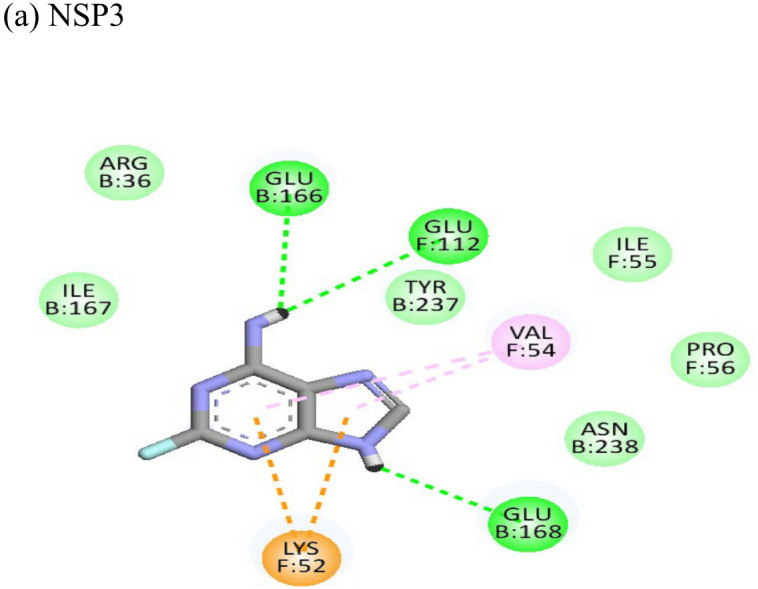	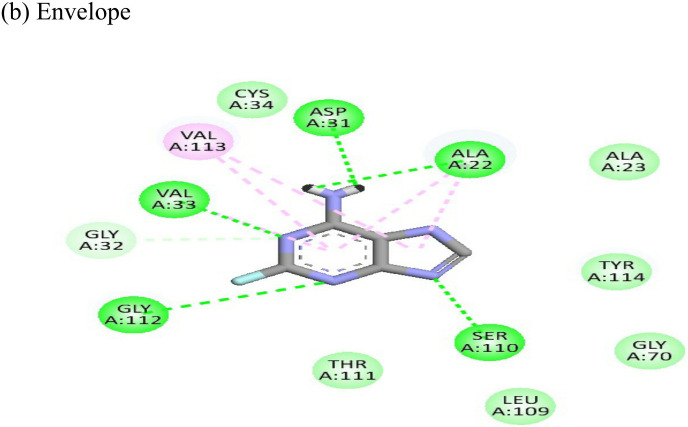	(a)-6.966 (b)-2.958	-37.69 -25.51	-11.091 -5.632
Doxorubicin	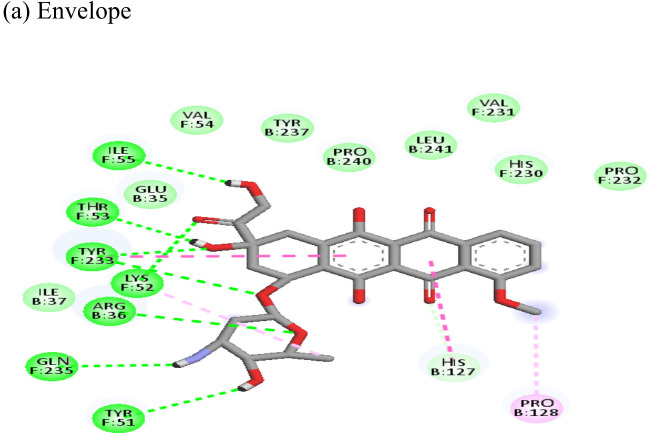	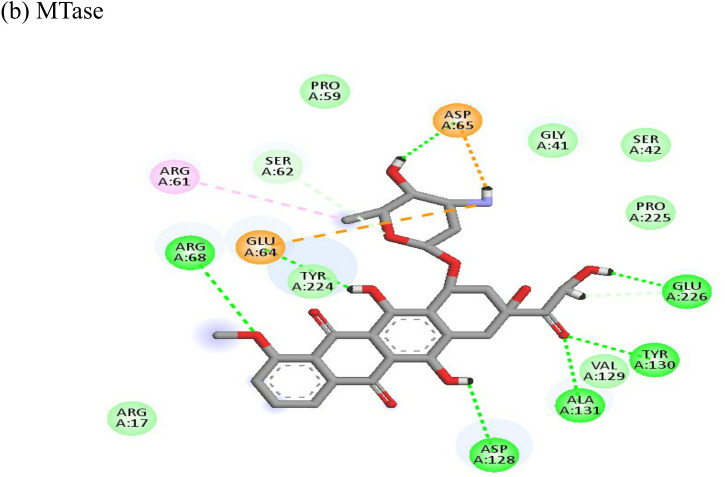	(a)-4.76 (b)-6.069 (c)-3.547	-77.88 -77.55 -77.21	-13.179 -15.343 -13.297
	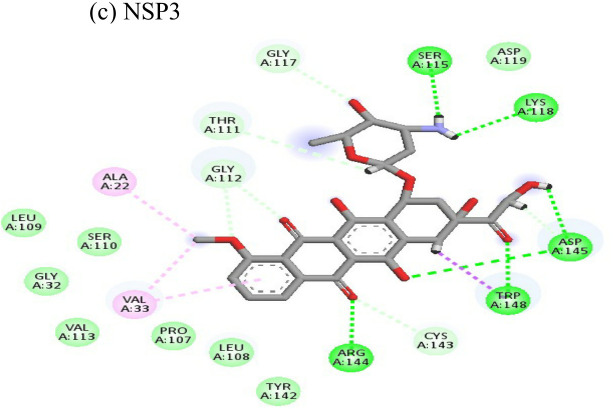			
Felbiac	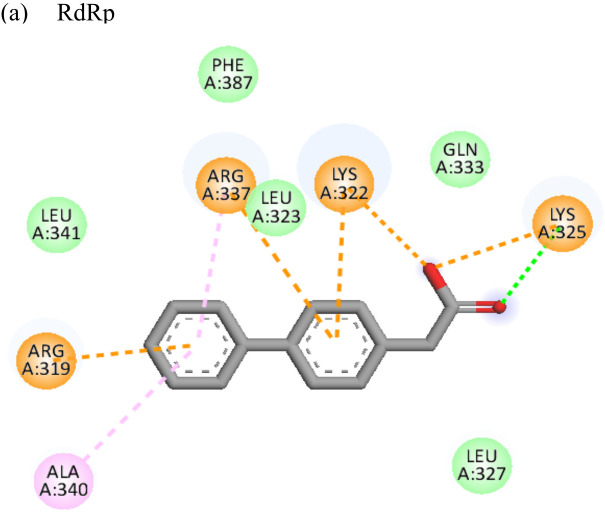	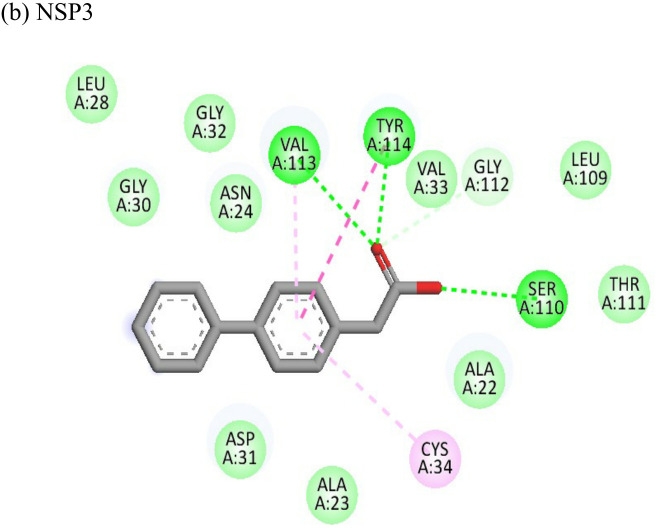	(a)-4.332 (b)-6.115	-44.3 -39.72	-11.74 -10.529
Metyrapone	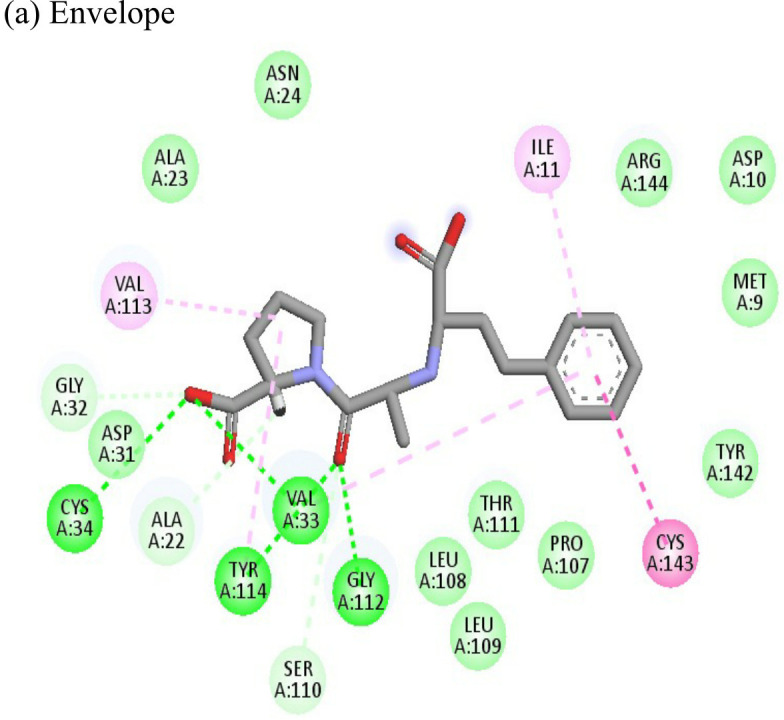	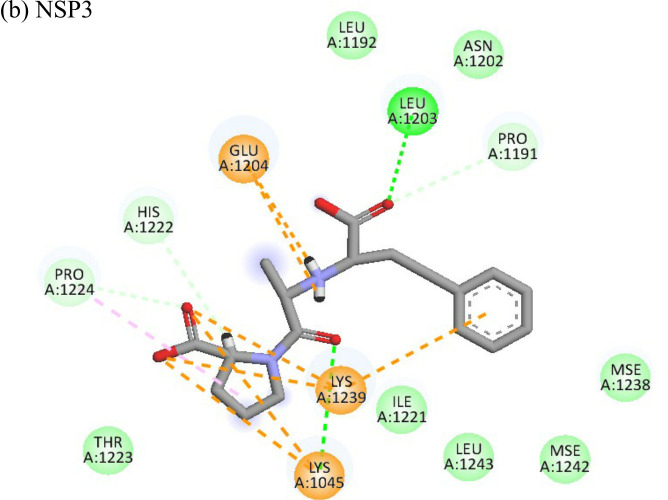	(a)-4.358 (b)-6.498	-51.12 -50.81	-12.521 -13.255
Enalprilat	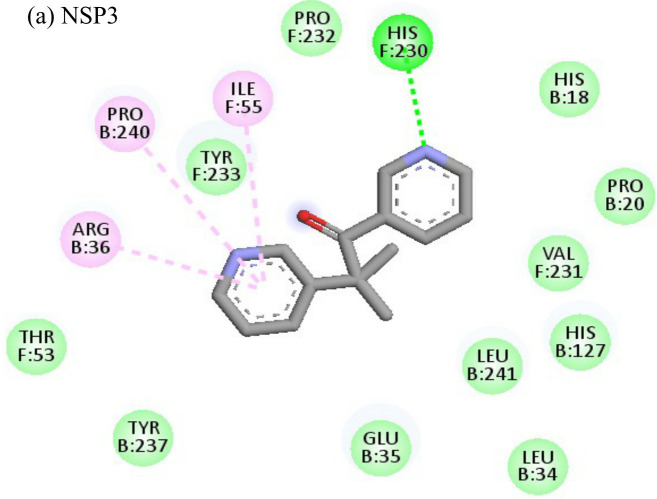	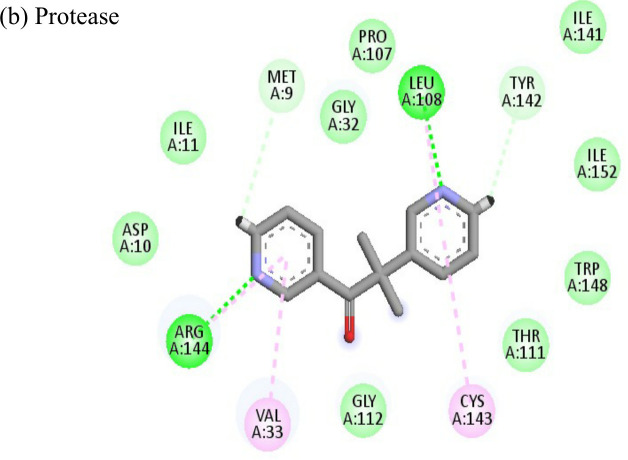	(a)-7.476 (b)-4.02	-60.97 -55.33	-14.452 -11.234
Emetine	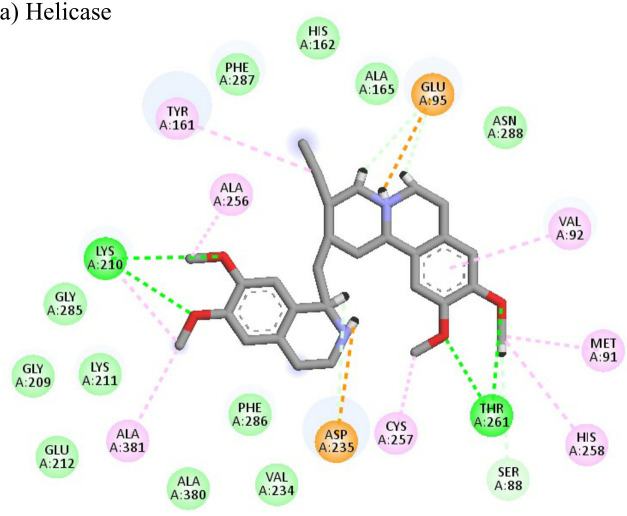	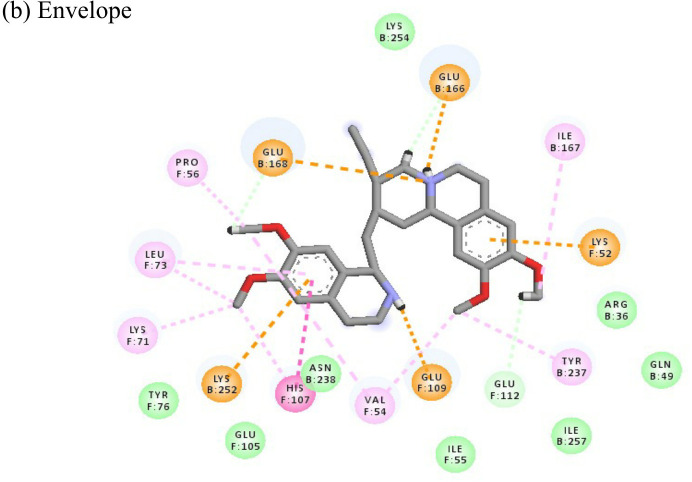	(a)-4.086 (b)-4.238	-74.25 -71.81	
Resveratrol	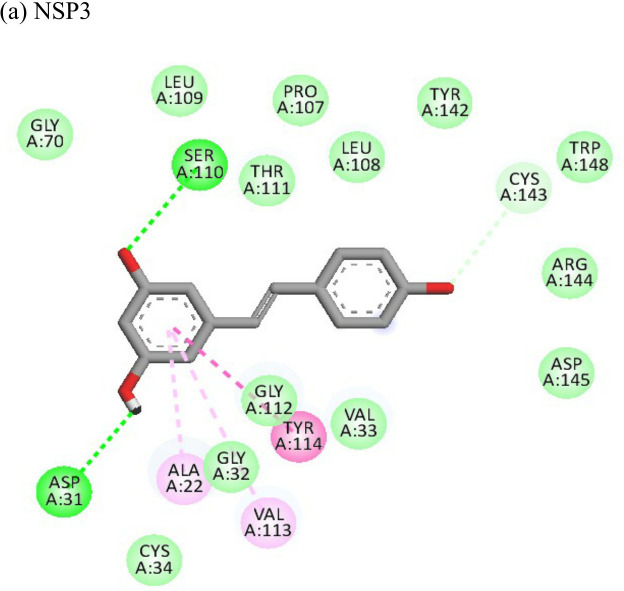	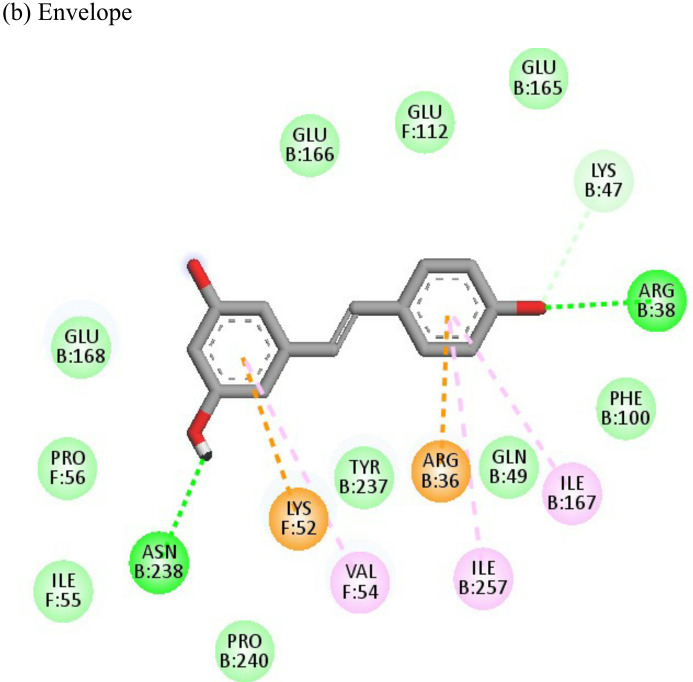	(a)-6.22 (b)-4.675	-56.45 -47.43	-14.72 -11.734
Lombubivir	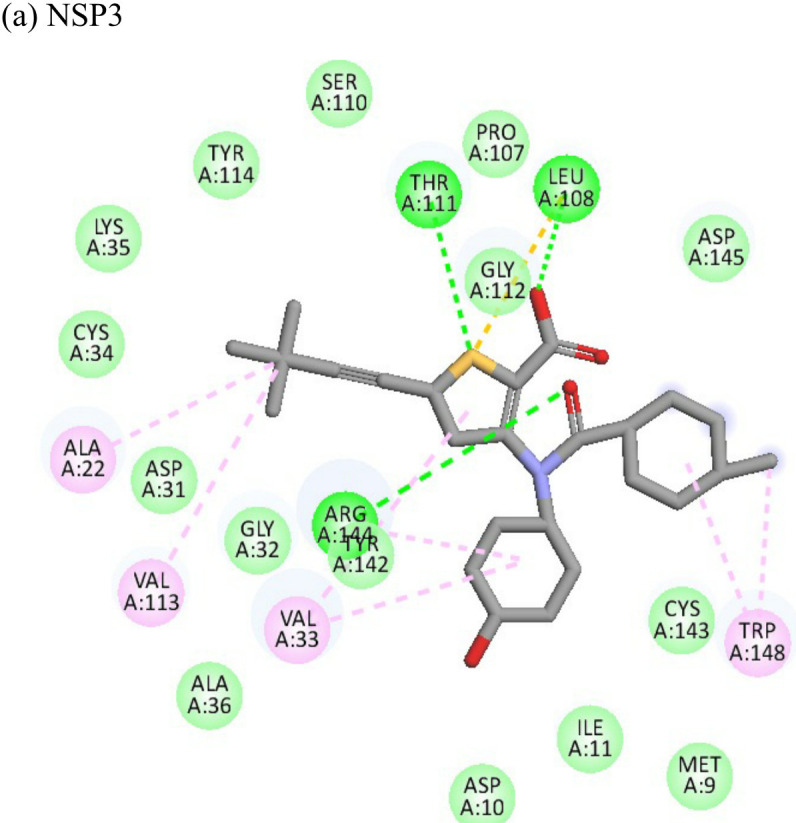	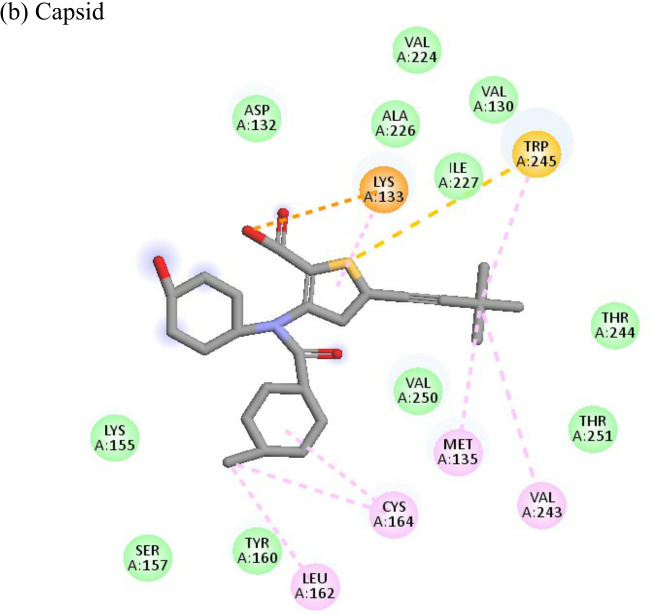	(a)-5.573 (b)-4.759	-81.13 -78.83	-18.293 -17.33

**Table 5:** Computational analysis of MM/GBSA binding free energies of compounds represented in kcal/mol.

	MMGBSA Values	Capsid (D-Score 0.959)	E-protein (D-Score 1.021)	Methyl transferase (D-Score 1.066)	Protease (D-Score 0.983)	Helicase (D-Score 1.012)	RdRp (D-Score 0.833)	NSP3 (D-Score 0.937)
2-Fluroadenine	dG Bind	-21.15	-25.51	-16.96	-18.29	-26.69	-24.13	-37.69
	dG Bind Coulomb	-17.82	-20.99	-14.23	-15.09	-18.05	-16.09	-33.08
	dG Bind Covalent	0.58	0	0	0	1.4	0.92	3.87
	dG Bind Hbond	-0.92	-2.62	-4.15	-4.84	-1.01	-1.76	-1.96
	dG Bind Lipo	-2.62	-3.59	-1.19	-1.93	-3	-2.21	-3.79
	dG Bind Packing	-0.84	-0.13	0	0	-0.39	-1.32	-0.23
	dG Bind SelfCont	0	0	0	0	0	0	0
	dG Bind Solv GB	14.6	21.5	18.64	18.18	15.08	14.03	17.28
	dG Bind vdW	-14.12	-19.68	-16.02	-14.62	-20.74	-17.7	-19.77
Doxo	dG Bind		-77.88	-77.21	-48.63	-69.62	-51.51	-71.55
	dG Bind Coulomb		-38	-25.38	36.49	-27.56	-36.38	-26.99
	dG Bind Covalent		0	0	0	5.33	0.94	2.94
	dG Bind Hbond		-1.55	-3.13	-1.83	-3.99	-2.38	-1.55
	dG Bind Lipo		-36	-30.94	-26.09	-29.55	-19.32	-29.97
	dG Bind Packing		-0.1	-3.39	0	0	-0.54	-0.59
	dG Bind SelfCont		0	0	0	0	0	0
	dG Bind Solv GB		48.54	37.2	-31.99	29.27	44.44	24.53
	dG Bind vdW		-50.77	-51.57	-25.2	-43.13	-38.28	-39.92
Felbinac	dG Bind	-30.85	-18.24	-29.15	-37.44	-26.86	-44.3	-39.72
	dG Bind Coulomb	-51.61	21.42	-53.11	-78.85	-24.95	-34.95	-34.53
	dG Bind Covalent	0	0	0	0	2.03	-0.37	0.59
	dG Bind Hbond	-3.98	-0.59	-3.74	-1.3	-2.5	-2.94	-1.83
	dG Bind Lipo	-10.34	-23.59	-16.43	-20.43	-21.49	-20.19	-19.93
	dG Bind Packing	0	-1	-0.03	0	0	-1.21	-0.11
	dG Bind SelfCont	0	0	0	0	0	0	0
	dG Bind Solv GB	48.36	9.39	68.45	89.39	42.65	35.43	43.54
	dG Bind vdW	-13.28	-23.87	-24.3	-26.25	-22.6	-20.08	-27.45
Metyrapone	dG Bind	-33.03	-51.12	-35.31	-40	-43.66	-22.13	-50.81
	dG Bind Coulomb	-1.45	-16.64	-11.79	-7.28	-14.08	0.55	-4.77
	dG Bind Covalent	0	0	0	0	5.3	-0.48	1.46
	dG Bind Hbond	-0.24	-0.49	-0.24	-0.65	-0.65	-0.57	-0.5
	dG Bind Lipo	-15.77	-23.56	-15.99	-24.65	-20.86	-12.39	-26.59
	dG Bind Packing	-0.23	0	-0.39	0	-0.55	-0.04	-0.14
	dG Bind SelfCont	0	0	0	0	0	0	0
	dG Bind Solv GB	6.15	12.34	19.02	11.68	17.61	8.82	13.27
	dG Bind vdW	-21.49	-22.77	-25.93	-19.09	-30.43	-18.02	-33.53
Enalprilat	dG Bind	-48.05	-39.25	-43.62	-55.33	-52.54	-36.06	-60.97
	dG Bind Coulomb	-19.5	26.99	-45.41	-82.79	-17.88	23.62	-60.02
	dG Bind Covalent	-17.82	0	0	0	0.35	0.83	7.8
	dG Bind Hbond	-3.85	-3.99	-3.44	-3.94	-3.59	-7.26	-1.74
	dG Bind Lipo	-41.36	-19.04	-31.23	-25.3	-26.6	-8.56	-41.88
	dG Bind Packing	-0.51	0	-1.34	0	0	-0.07	0
	dG Bind SelfCont	0	0	0	0	0	0	0
	dG Bind Solv GB	29.95	-7.08	73.14	92.77	35.17	-17.92	72.88
	dG Bind vdW	-12.78	-36.12	-35.34	-36.07	-39.98	-26.7	-38.01
Emetine	dG Bind	-21.15	-71.81	-64.17		-74.25	-46.8	
	dG Bind Coulomb	-17.82	-49.99	-27.66		-42.09	-61.18	
	dG Bind Covalent	0.58	0	0		2.8	6.11	
	dG Bind Hbond	-0.92	-1.43	-1.08		-2.04	-0.74	
	dG Bind Lipo	-2.62	-37.53	-46.36		-42.46	-30.46	
	dG Bind Packing	-0.84	-0.12	0		0	-0.02	
	dG Bind SelfCont	0	0	0		0	0	
	dG Bind Solv GB	14.6	70.4	52.61		52.93	78.66	
	dG Bind vdW	-14.12	-53.14	-41.69		-43.4	-39.16	
Resveratrol	dG Bind	-41.58	-47.43	-44.71	-28.41	-42.82	-28.81	-56.45
	dG Bind Coulomb	-2.92	-25.97	-28.75	-16.29	-25.59	-10.57	-17.17
	dG Bind Covalent	0	0	0	0	3.21	2.22	4.73
	dG Bind Hbond	-0.3	-1.82	-2	-0.68	-1.75	-0.37	-0.64
	dG Bind Lipo	-30.19	-26.66	-21.37	-23.29	-19.02	-14.99	-26.92
	dG Bind Packing	-0.32	-0.3	-2.81	-0.6	-0.11	-0.12	-0.15
	dG Bind SelfCont	0	0	0	0	0	0	0
	dG Bind Solv GB	15.37	30.01	34.5	26.58	25.09	16.59	19.3
	dG Bind vdW	-23.2	-22.69	-24.29	-14.13	-24.66	-21.57	-35.6
Lombubivir	dG Bind	-78.83	-38.07	-55.91	-52.47	-61.56	-49.76	-81.13
	dG Bind Coulomb	-17.33	-14.11	-20.42	-76	-32.11	-13.08	-23.97
	dG Bind Covalent	0	0	0	0	13.61	2.97	11.84
	dG Bind Hbond	-0.13	-1.87	-0.05	-1.57	-2.83	-8.45	-1.01
	dG Bind Lipo	-54.11	-23.93	-44.84	-32.76	-55.9	-22.85	-62.33
	dG Bind Packing	0	0	0	0	0	-0.01	-0.49
	dG Bind SelfCont	0	0	0	0	0	0	0
	dG Bind Solv GB	31.69	34.85	54.87	83.6	64.02	17.35	42.41
	dG Bind vdW	-38.94	-33.02	-45.48	-25.74	-48.36	-25.69	-47.6

#### 3.9.1 2-Fluroadenine

2-Fluoroadenine showed highest negative binding energy of -37.69 kcal/mol with the NSP3 protein. The 2D interaction diagram (**Table 4**) shows that it formed an alkyl bond with the residue val54, a pi cation bond with lys52 and conventional hydrogen bonds with residues glu168, glu112, and glu166. It showed the second highest binding energy (-25.51 kcal/mol) with the CHIKV envelope protein forming 5 conventional hydrogen bonds with residues ala22, asp31, val33, ser110 and gly112 following 4 alkyl bonds with two ala22 and two asp31 respectively. This indicates a possible inhibitory mechanism of action of the drug 2-fluroadenine against the NSP3 domain and/or the envelope protein. The ligand efficiency for 2-fluroadenine as a ligand, was further found to be better for NSP3 as compared to the envelope (-11.091kcal/mol with NSP3 and -5.632 kcal/mol with envelope protein).

#### 3.9.2 Doxorubicin

Interactions of Doxorubicin with the envelope protein showed a binding energy of -77.88 kcal/mol, forming one pi-pi T-shaped bond with residues his127 and Tyr 233, one alkyl bond with lys52, and eight conventional hydrogen bonds with ile55, thr53, tyr233, lys 52, arg36, tyr51, and gln235 (**Table 4**). Doxorubicin showed almost equal binding energies of -77.55 kcal/mol and -77.21 kcal/mol with methyl transferase and NSP3 targets, respectively. In case of the former, it forms five conventional hydrogen bonds with asp128, ala131, tyr139, glu226, arg68, alkyl bond with arg61; and a pi-cation with asp65 and glu64 residues. Similarly, doxorubicin forms six hydrogen bonds with aspartate (asp145), trp148, arg144, lys118, and ser115 residues, as well as two alkyl bonds with residues ala22 and val33. Thus, doxorubicin drug interactions with residues present in the catalytic site of the enzyme indicate its possible inhibitory mechanism against the envelope, methyl transferase, and NSP3 CHIKV proteins, and having high ligand efficiencies of -13.179, -15.343, and -13.297 kcal/mol, respectively.

#### 3.9.3 Felbinac

The molecular docking analysis of the drug felbinac with CHIKV targets revealed that felbinac had strong interactions with the RdRp and NSP3 macro domain (**Table 4**). Felbinac formed pi-cation bond with residues arg337, arg319, lys322 and lys325 following pi-alkyl bond with residues arg319, arg340 and arg337. Felbinac showed a binding affinity of -39.72 kcal/mol forming strong hydrogen bonds with val113, tyr114 and ser110.

#### 3.9.4 Metyrapone

Metyrapone showed strong interactions with envelope and NSP3 having binding energy values -51.12 and -50.81 kcal/mol respectively. Ligand efficiency with the envelope was -4.358 and -6.498 kcla/mol with NSP3 protein. It formed strong hydrogen bond with residue his230 and alkyl bonds with residues ile55, pro240 and arg36 (**Table 4**).

#### 3.9.5 Enalaprilat

Enalaprilat displayed a strong binding energy of -60.97 kcal/mol with the NSP3 micro domain (**Table 3**). It interacted with residues tyr114, val33, gly112, cys34 forming four hydrogen bonds, four alkyl interactions with val113, tyr114, ile11 and val33 and one pi-pi T shaped bond with cys143. Enalaprilat docked to the protease with a binding energy of -55.33 kcal/mol. It interacted with residues leu1203 and lys1025 by forming two hydrogen bonds. SiteMap was used to predict the binding pocket of the protease. The docking studies identified strong interactions with this predicted target in case of emetine (**Table 4**).

#### 3.9.6 Emetine

Following the docking analysis among the seven CHIKV target proteins, emetine showed highest binding energy with CHIKV helicase and envelope protein showing binding energies of -74.25 and -71.81 kcal/mol respectively. Emetine displayed a ligand efficiency of -4.086 kcal/mol to the CHIKV helicase. The 2D interactions of the drug with the helicase revealed four hydrogen bonds involving residues lys210 and thr261 which are in the vicinity of the highly conserved residues. It interacted with residues ala381, cys257, his258, met91, val92, ala256 and tyr161 forming eight pi-alkyl interactions. Envelope protein interacts with tyr237, val54, ile167, leu73, lys71, pro56 forming alkyl bonds; his107 forms pi-pi T shaped residues (**Table 4**).

#### 3.9.7 Resveratrol

Resveratrol showed the highest docking score of -6.22 with NSP3 domain followed by -4.675 docking score with envelope protein. It formed strong hydrogen bonds with residues ser110 and asp31. It formed one pi T shaped bond with tyr114. Resveratrol formed two hydrogen bonds with residues arg38 and asn238 of the envelope protein. (**Table 4**). Highest ligand efficiencies of -14.72 kcal/mol and -11.734 kcal/mol was observed with NSP3 and envelope, respectively.

#### 3.9.8 Lomibuvir

The ligand efficiency of lomibuvir with NSP3 and capsid were -18.293 kcal/mol and -17.33 kcal/mol respectively. It showed highest binding energy of -81.13 kcal/mol with NSP3 domain forming strong hydrogen bonds with thr111, leu108 and arg144, pi-alkyl bonds with thr148, val33, val113, ala22 and leu108. Following the highest binding energy with NSP3 domain lomibuvir showed strong interactions with the capsid protein having a binding energy -78.83 kcal/mol (**Table 4**).

#### 3.9.9 Temsirolimus

In the HTVS protocol, Temsirolimus failed to fit the pockets created by SiteMap for all the chikungunya targets, However, when individual target dockings were undertaken, it could fit in the envelope protein of CHIKV, showing a docking score of -4.6 (**Figure S7**).

## 4 Discussion

CHIKF has been a serious public health problem in tropical and sub-tropical regions in the world and the only preventive measure available for CHIKV is the control of mosquitoes. As of now, there is no effective licensed therapeutics or vaccines available for the treatment of CHIK fever. Several drug candidates have been tested for their antiviral activity against CHIKV to date. The drug development process is lengthy and takes years to complete before a drug can be used by the intended population. Drug repurposing/redirecting/repositioning or re-profiling, can be used to get around this problem. Towards the search to discover newer CHIK viral targets and mechanisms for the development of novel antivirals, in the present study, we investigated the anti-CHIKV activity of fourteen FDA-approved drugs. Some of these drugs were identified as potential drug candidates against dengue, another mosquito-borne disease (Punekar et al., 2022) which mimics CHIKF in symptoms (**Table 3**).

Among the fourteen drugs, nine drugs showed anti-CHIKV activity. Temsirolimus tops the list of anti-CHIKV drugs with a SI of 85.1 and 65.4 for prophylactic and therapeutic activities. The study results revealed that temsirolimus reduced CHIKV tire by 4-5 log_10_ titres compared to VC and anti-CHIKV activity was confirmed by different assays which measure, infectious virus particle titre, viral RNA copy number, percent of infected cells and viral antigen levels. Temsirolimus is an anti-cancer agent used for the treatment of advanced renal cell cancer and is an inhibitor of the mammalian target of rapamycin (mTOR). mTOR is a serine/threonine kinase and a decrease in mTOR activity affects protein synthesis and cell cycle (Malizzia and Hsu, 2008). Temsirolimus has been reported to possess anti-SARS CoV-2 and anti-HBV activities (Saso et al., 2018; Mullen et al., 2021). While the *in silico* HTVS study, showed that among the nine compounds, temsirolimus displayed an overall ineffective pharmacological profile for chikungunya targets, not displaying suitable ligand complementarity with the SiteMap defined regions. However there is a possibility that among the CHIKV targets, it may interact with the envelope glycoproteins as noted from the moderate docking score. Hence, it is possible that the mode of action of temsirolimus inhibiting CHIKV replication, is by targeting both the host pathways and the viral envelope glycoprotein.

2 Fluoroadenine and doxorubicin were found to be most effective with maximum inhibition in CHIKV in pre and posttreatment conditions. *In-silico* docking analysis suggested that 2-fluoroadenine interacted with NSP3 and envelope glycoprotein while doxorubicin interacted with envelope glycoprotein, NSP1 methyltransferase protein and NSP3. 2-Fluoroadenine is a nucleoside analogue and acts as an antimetabolite Several studies reported the antineoplastic property of doxorubicin against breast, lung, non-Hodgkin’s, and Hodgkin’s lymphoma, gastric, thyroid multiple myeloma, sarcoma, ovarian, and paediatric cancers (Thorn et al., 2011). Previous studies also explain the efficacy of doxorubicin against several viruses (Johansson et al., 2006; De Palma et al., 2007; Coelmont et al., 2009; Kaptein et al., 2010; Al-Motawa et al., 2020). Doxorubicin and its derivatives showed significant inhibitory activities against dengue and yellow fever virus replication in *in vitro* studies. The major limitation of doxorubicin is, it is reported to cause cardiotoxicity. According to a study by Thorn et al., doxorubicin’s toxicity can be decreased while maintaining higher efficacy, and this could be achieved by experimenting with different derivatives or metabolites (Thorn et al., 2011). Therefore, more research on anthracyclines is required because they are the key to developing effective antiviral and anticancer drugs. Furthermore, doxorubicin exposure over a prolonged period of time (six weeks during cancer treatments) causes cardiotoxicity, whereas CHIKV fever only lasts a few days to a few weeks.

Resveratrol is an anti-inflammatory drug; earlier, it has been used as a repurposed drug against MERS-CoV infection (Lin et al., 2017). Our recent study reported that resveratrol possesses anti-DENV activity (Punekar et al., 2022). The present study revealed that resveratrol at 12.5 µM and 6.25 µM concentrations significantly inhibited the CHIKV virus. *In silico* studies revealed that it interacts with the NSP3 and envelope glycoprotein of the virus. Our study showed strong interactions of lomibuvir with NSP3 and capsid targets following the significant reduction in viral titre under posttreatment conditions. It is possible that lomibuvir demonstrates inhibition because of its NSP3 and capsid inhibition property. *In-silico* docking analysis revealed that lomibuvir can interact with NSP3 and capsid proteins of CHIKV. It showed an inhibitory effect at 6.25 µM concentration in the posttreatment condition. Emetine, the viral polymerase inhibitor, is a frequently used drug against malaria. It shows potential antiviral activity against diverse sets of DNA as well as RNA viruses including Zika, Ebola, HIV-1, Cytomegalovirus etc. (Andersen et al., 2019) Several virus-specific assays showed that emetine affects the synthesis of viral RNA and DNA as well as virus entry. Besides that, emetine can affect viral protein synthesis in mammalian cells by inhibiting the aminoacyl-sRNA 83 transfer reaction at the 40s subunit (Khandelwal et al., 2017). *In-silico* investigation suggested that emetine interacted with NSP2 and envelope glycoproteins with high binding energy. Our study showed an inhibitory effect at 200 µM, and 100 µM, concentrations in posttreatment conditions. Its polymerase inhibitor property may affect viral replication and hence the results against CHIKV under posttreatment conditions.

Metyrapone is an inhibitor of glucocorticoid synthesis and has a positive effect against Cushing’s syndrome (Daniel et al., 2015). The present study showed an inhibitory effect of metyrapone against CHIKV under pretreatment condition with a SI of 457.8. Felbinac is a non-steroidal anti-inflammatory drug used for the treatment of osteoarthritis. It showed an inhibitory effect under pretreatment condition with a SI of2205, .88. The drugs which exerted anti-CHIKV activity under pretreatment conditions had the highest SI compared to drugs which exerted anti-CHIKV activity under other conditions. These drugs might have prophylactic utility during outbreaks of CHIKV. Enalaprilat, which is an ACE inhibitor, was also found to affect CHIKV under cotreatment condition. Virus attachment and entry assays revealed that the drug affects virus attachment rather than virus entry. Though *in-silico* studies suggested that enalaprilat interacts with non-structural proteins, the drug did not affect CHIKV titer post infection indicating that interaction may not affect non-structural proteins. Among the drugs that affect CHIKV titer post infection including temsirolimus, 2-fluoroadenine, doxorubicin, resveratrol, emetine and lomibuvir, all the drugs affected CHIKV titer even when added at 6 h time point at higher concentrations studied. The antiviral effect was maximum when the drugs were added at earlier time points which could be attributed to the rapid replicating nature of the virus. These drugs can affect both early and late phase of CHIKV life cycle. However, it may not be possible to indicate the actual phase of the viral life cycle affected by the drug using time of addition assays post infection since in a culture of infected cells, the life cycle of virus will be at different stages in different cells. Synchronizing the virus entry into cells followed by time of addition assays might help to identify the stage of the virus life cycle that is affected and needs further investigations. One of the limitations of the study is that host factors targeted by these drugs were not studied. Such investigation might provide more insight into the inhibitory mechanisms. Moreover, since these drugs affected both intra-and extracellular virus titer, it is possible that these drugs may not affect virus release.

Though most of drugs completely affected infectious virus particles (FFU), the inhibitory effect was not observed with regard to viral RNA levels. It is possible that these drugs may not be involved in inhibition of virus replication. Quantitative real-time RT-PCR is a more sensitive technique than FFU and can identify RNA from even non-infectious particles. As a result, unless there is a significant difference in the viral RNA titer, minor variations in viral RNA titer might not be detected in the findings of a quantitative real-time RT-PCR assay. The drugs which affected both FFU and viral RNA titer might inhibit viral RNA replication.

Among the drugs which showed anti CHIKV activity in the present study, resveratrol, doxorubicin, lomibuvir and enalaprilat were also reported to exert anti-DENV activity (Punekar et al., 2022). These drugs might be useful in regions where both viruses are endemic and need to be prioritized. Apart from the drugs with anti-DENV activity, temsirolimus can be taken forward since, the inhibitory effect against CHIKV was greater compared to other drugs. The results of the current investigation could serve as the foundation for *in vivo* studies that examine the possibility of treating chikungunya fever with FDA-approved drugs by drug repurposing.

## 5 Conclusions

The present study reports anti-CHIKV activity of select FDA-approved drugs, which can be further repurposed against CHIKV. The drugs with both anti-DENV and anti-CHIKV activity can be prioritized for further *in vivo* validation studies in small animals and subsequently trials for repurposing against these viruses.

## Data availability statement

The original contributions presented in the study are included in the article/**Supplementary Material**. Further inquiries can be directed to the corresponding authors.

## Author contributions

Conceived and designed the experiments: SC, DP, and KA; Performed the experiments: BK, GA, PP, MP, MK and KS; Analyzed the data: BK, GA, PP, MP, MK, KS, KA, DP, and SC; Wrote the paper: BK, MP, KS, KA, DP, and SC. All authors contributed to the article and approved the submitted version.

## Supplementary material

Supplementary Figure 1 Cytotoxic evaluation of selected drugs on Vero CCL-81 cells using MTT assay

Supplementary Figure 2 Evaluation of percent inhibition and IC50 value. For finding out the IC50 value of the following drugs i.e. Temsirolimus (pretreatment) **(A)**, Temsirolimus (posttreatment) **(B)**, 2-fluoroadenine (pretreatment) **(C)**, 2-fluoroadenine (posttreatment) **(D)**, Doxorubicin (pretreatment) **(E)**, Doxorubicin (posttreatment) **(F)**, Felbinac (pretreatment) **(G)**, Metyrapone (pretreatment) **(H)**, Enalaprilat (cotreatment) **(I)**, Emetine (posttreatment) **(J)**, Resveratrol **(K)** (posttreatment), Lomibuvir (posttreatment) **(L)**, percent inhibition of virus titre was calculated with reference to virus titre in VC and IC50 was calculated using non-linear regression analysis. All the values are represented as mean percent inhibition ± SE using VC as reference. ****p < 0.0001 vs VC.

Supplementary Figure 3 Effect of different drugs on CHIKV RNA levels, quantified by qRT-PCR under different treatment conditions.Vero CCL–81 cells were pre, co and posttreated with different concentrations of drugs i.e. Temserolimus **(A)** 2-Fluroadenine **(B)** Doxorubicin **(C)** Felbinac **(D)** Metyrapone **(E)** Enalprilat **(F)** Ementine **(G)** Resveratrol **(H)** Lombubivir **(I)**. After 24 h incubation, the plates were freezed and the culture filtrates were used for the qRT-PCR. Total RNA was isolated and CHIKV RNA was detected by measuring E3 RNA copies by real-time RT-PCR.Results were expressed as mean log_10_ viral RNA copies/ml ± SE and presented logarithmically. All the treatment groups were compared with a virus control (VC) group (without treatment). ****p < 0.0001, ***p < 0.001 vs. VC.

Supplementary Figure 4 Effect of different drugs added at 0 h time point post infection on the titer of extracellular and intracellular virus particles. Infected Vero CCL-81 cells were treated with different concentrations of drugs i.e. Temsorilmus **(A)**, 2-Flouroadenine **(B)**, Doxorubicin **(C)**, Emetine **(D)**, Resveratrol **(E)** and Lomibuvir **(F)** immediately post infection and incubated for 24 h post infection. Post incubation, culture supernatant and cells were separately assessed for virus titer using FFU assay. The outcomes are expressed as mean log_10_ foci forming unit/ml ± standard error. All the treatment conditions were compared with the VC. *p<0.05, **p<0.01, ***p<0.005, ****p<0.0001.

Supplementary Figure 5 Effect of different drugs added at 3 h time point post infection on the titer of extracellular and intracellular virus particles. Infected Vero CCL-81 cells were treated with different concentrations of drugs i.e. Temsorilmus **(A)**, 2-Flouroadenine **(B)**, Doxorubicin **(C)**, Emetine **(D)**, Resveratrol **(E)** and Lomibuvir **(F)** at 3h post infection and incubated for 24 h post infection. Post incubation, culture supernatant and cells were separately assessed for virus titer using FFU assay. The outcomes are expressed as mean log_10_ foci forming unit/ml ± standard error. All the treatment conditions were compared with the VC. *p<0.05, **p<0.01, ***p<0.005, ****p<0.0001.

Supplementary Figure 6 Effect of different drugs added at 6 h time point post infection on the titer of extracellular and intracellular virus particles. Infected Vero CCL-81 cells were treated with different concentrations of drug i.e. Temsorilmus **(A)**, 2-Flouroadenine **(B)**, Doxorubicin **(C)**, Emetine **(D)**, Resveratrol **(E)** and Lomibuvir **(F)** at 6 h post infection and incubated for 24 h post infection. Post incubation, culture supernatant and cells were separately assessed for virus titre using FFU assay. The outcomes are expressed as mean log_10_ foci forming unit/ml ± standard error. All the treatment conditions were compared with the VC. *p<0.05, **p<0.01, ***p<0.005, ****p<0.0001.

Supplementary Figure 7 Docking of temsirolimus to envelope protein and the resulting D-Score.
