# Corrigendum: Drug repurposing approach against chikungunya virus: an *in vitro* and *in silico* study

**DOI:** 10.3389/fcimb.2023.1226054

**Published:** 2023-06-23

**Authors:** Bhagyashri Kasabe, Gunwant Ahire, Poonam Patil, Madhura Punekar, Kusuma Sai Davuluri, Mahadeo Kakade, Kalichamy Alagarasu, Deepti Parashar, Sarah Cherian

**Affiliations:** ^1^ Bioinformatics Group, Indian Council of Medical Research (ICMR)-National Institute of Virology, Pune, Maharashtra, India; ^2^ Dengue & Chikungunya Group, Indian Council of Medical Research (ICMR)-National Institute of Virology, Pune, Maharashtra, India

**Keywords:** chikungunya virus (CHIKV), drug repurposing, structural and non-structural proteins, *in silico* screening, *in vitro* validation

In the published article, there was an error in **Table 4** as published. The target for metyrapone was listed twice while the target for lomibuvir was missed. The corrected **Table 4** and its caption [Molecular docking interactions of the nine FDA approved drugs with CHIKV structural and non-structural proteins based on the binding affinity values and best pose] appear below.

**Table 4:** Molecular docking interactions of the nine FDA approved drugs with CHIKV structural and non-structural proteins based on the binding affinity values and best pose.

Compound	Potential binding viral targets		Docking score	Binding energy (kcal/mol)	Ligand Efficiency (kcal/ mol)
2-Fluroadenine	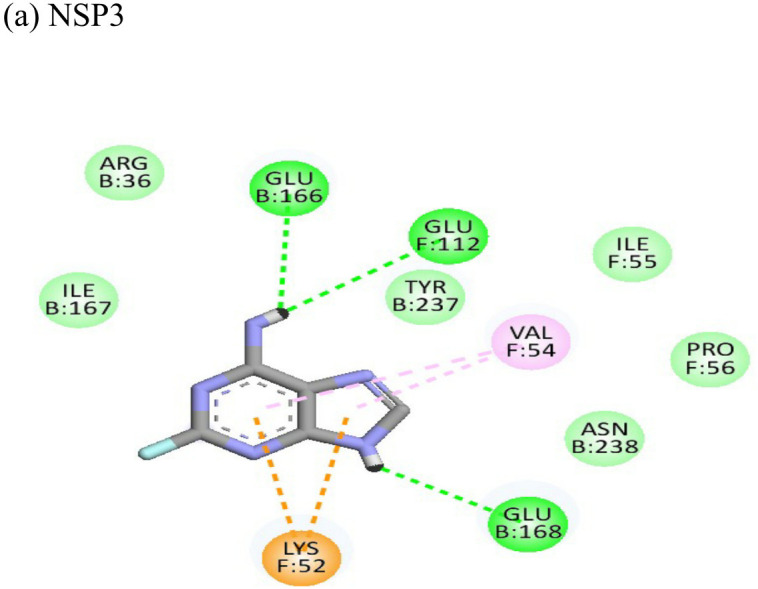	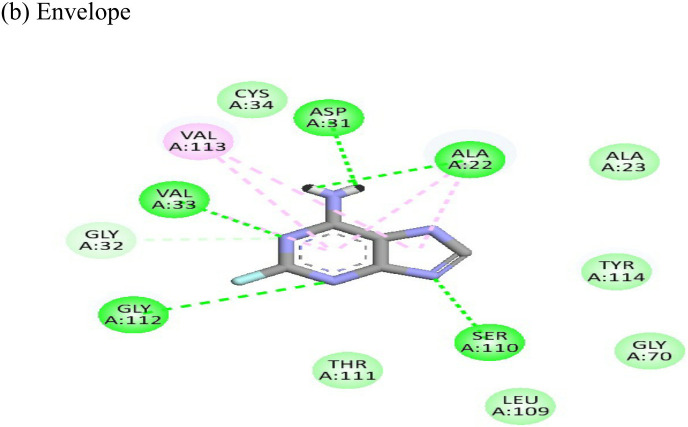	(a)-6.966 (b)-2.958	-37.69 -25.51	-11.091 -5.632
Doxorubicin	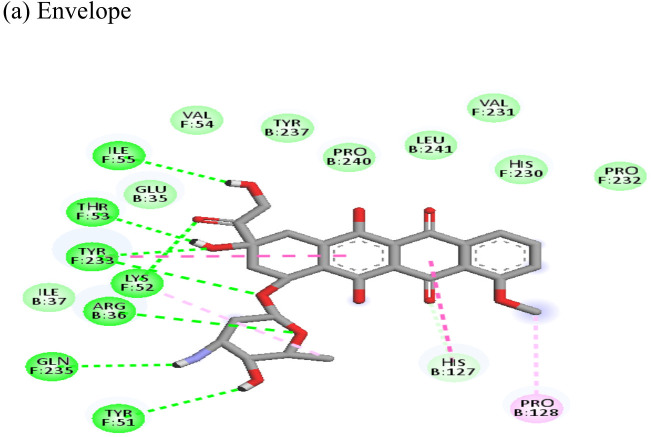	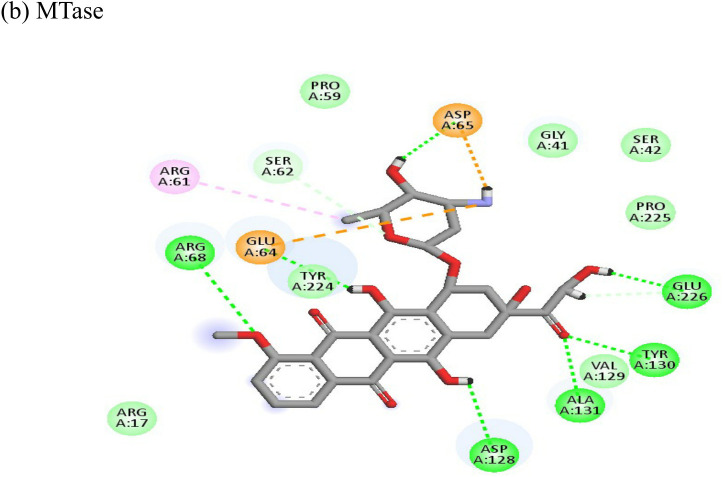	(a)-4.76 (b)-6.069 (c)-3.547	-77.88 -77.55 -77.21	-13.179 -15.343 -13.297
	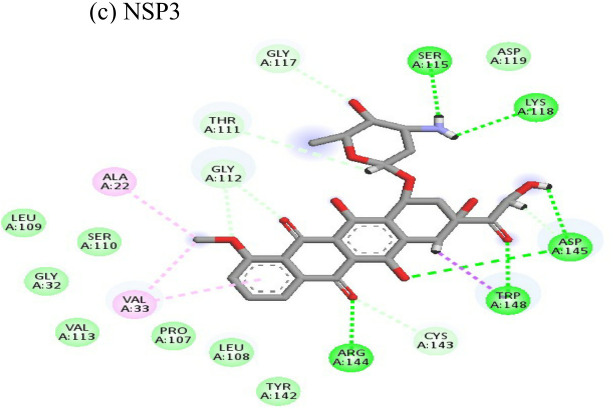				
Felbiac	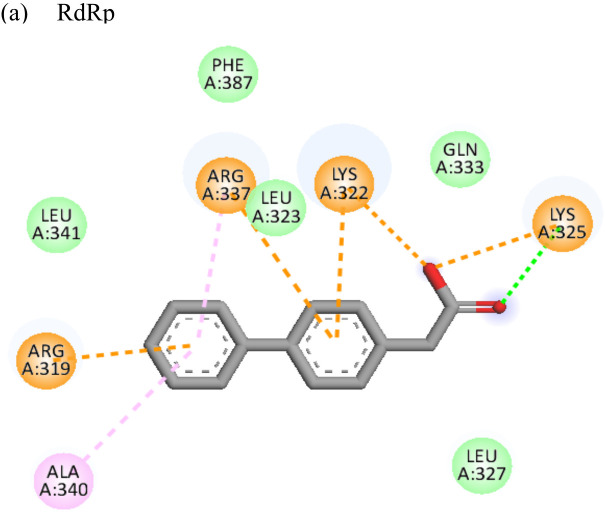	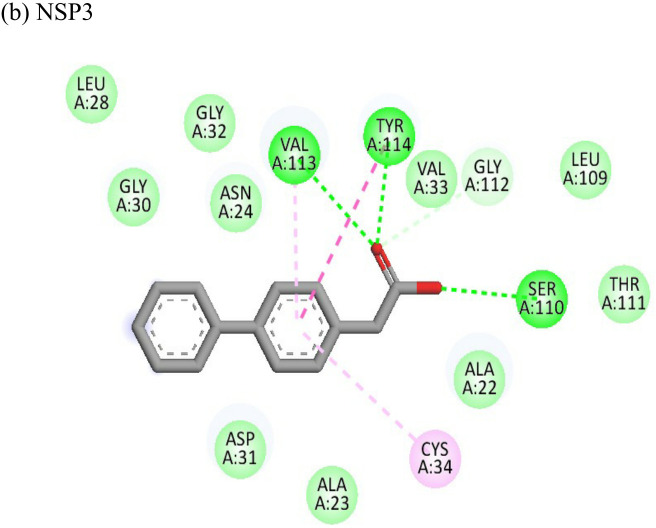	(a)-4.332 (b)-6.115	-44.3 -39.72	-11.74 -10.529
Metyrapone	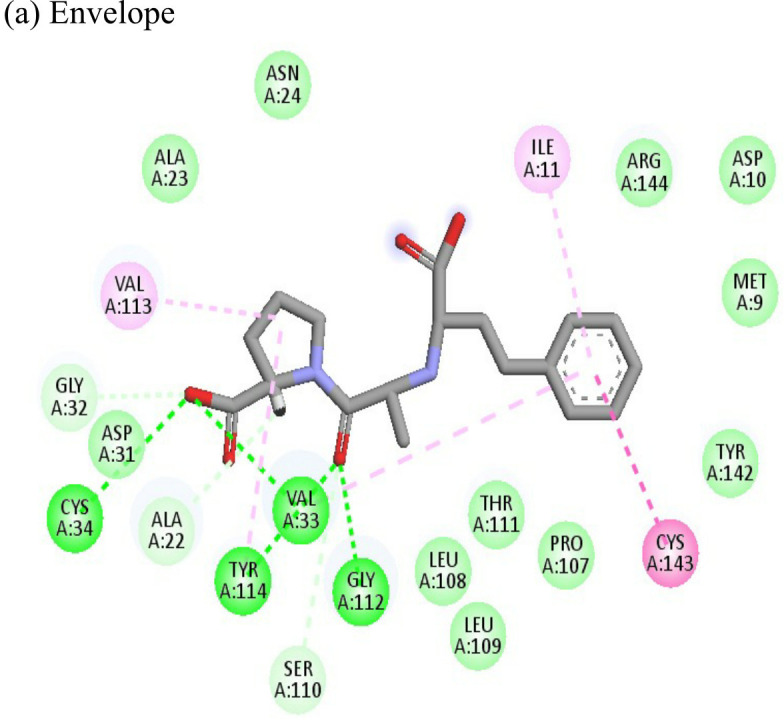	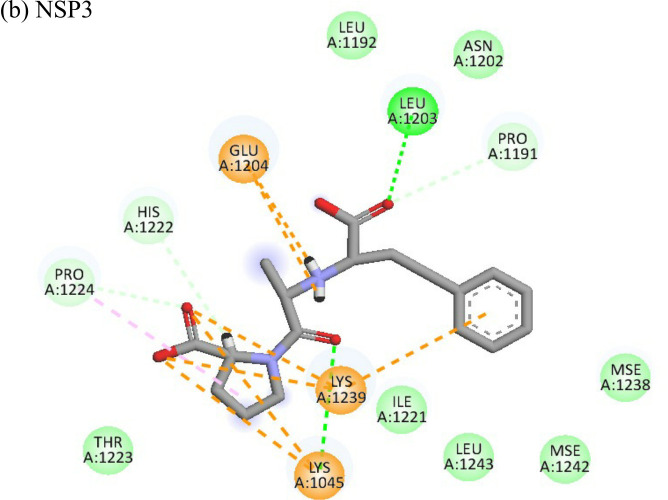	(a)-4.358 (b)-6.498	-51.12 -50.81	-12.521 -13.255
Enalprilat	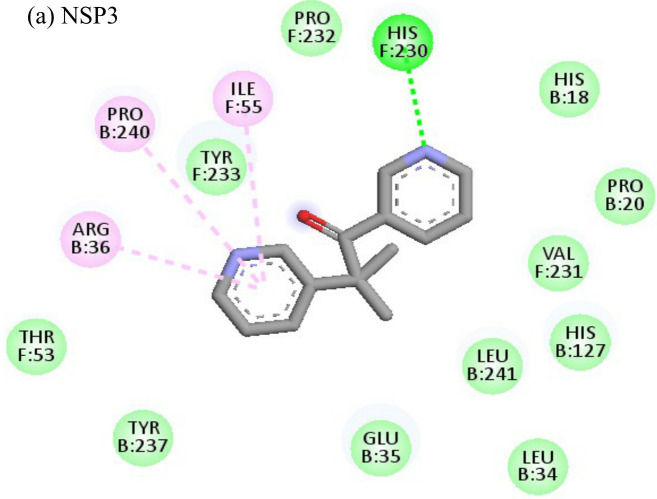	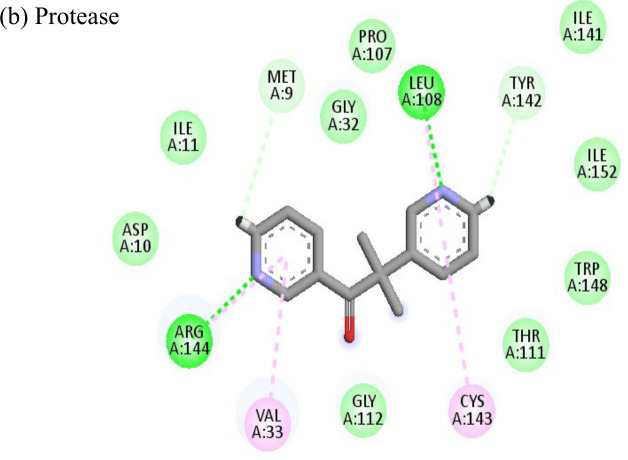	(a)-7.476 (b)-4.02	-60.97 -55.33	-14.452 -11.234
Emetine	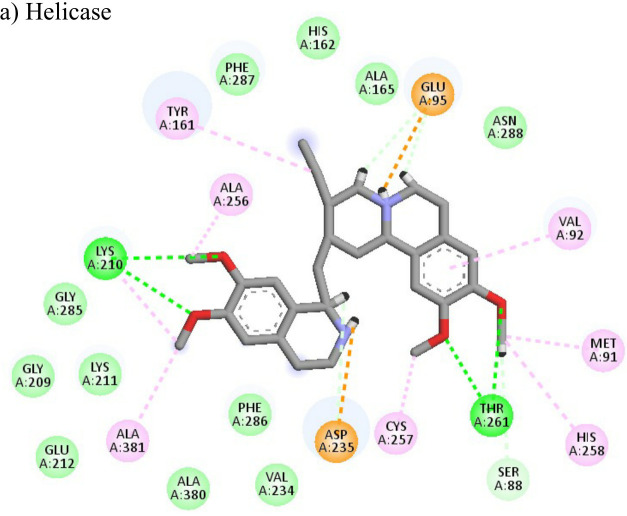	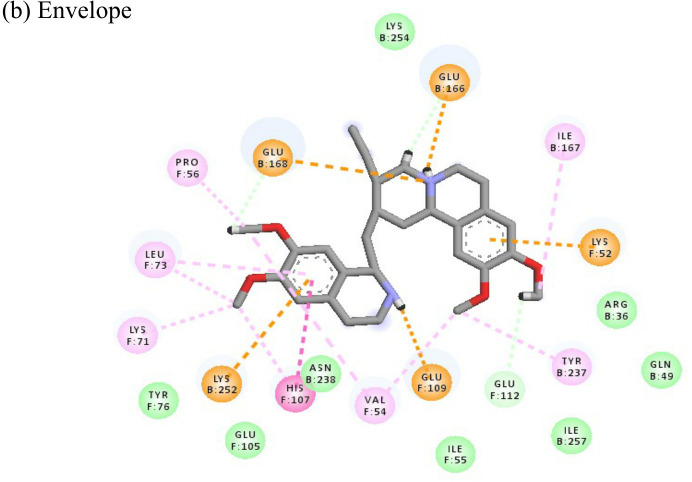	(a)-4.086 (b)-4.238	-74.25 -71.81	
Resveratrol	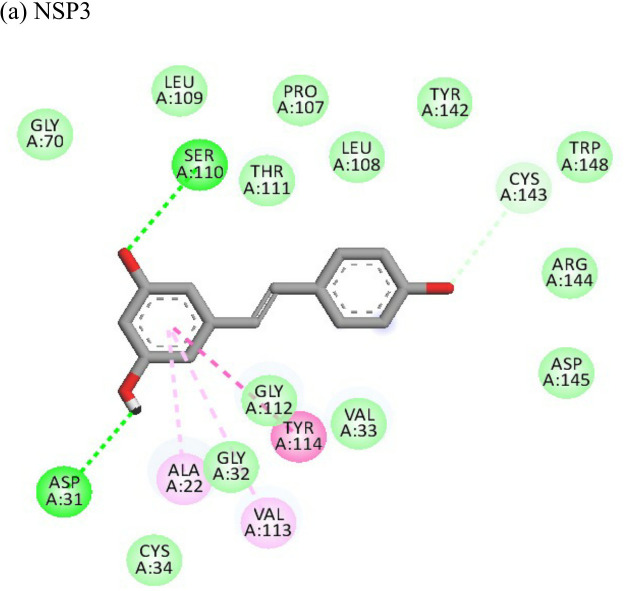	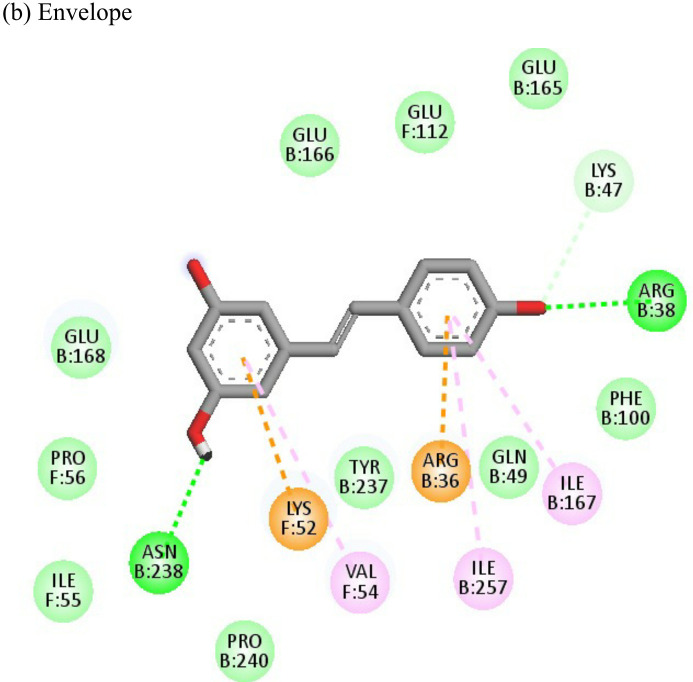	(a)-6.22 (b)-4.675	-56.45 -47.43	-14.72 -11.734
Lombubivir	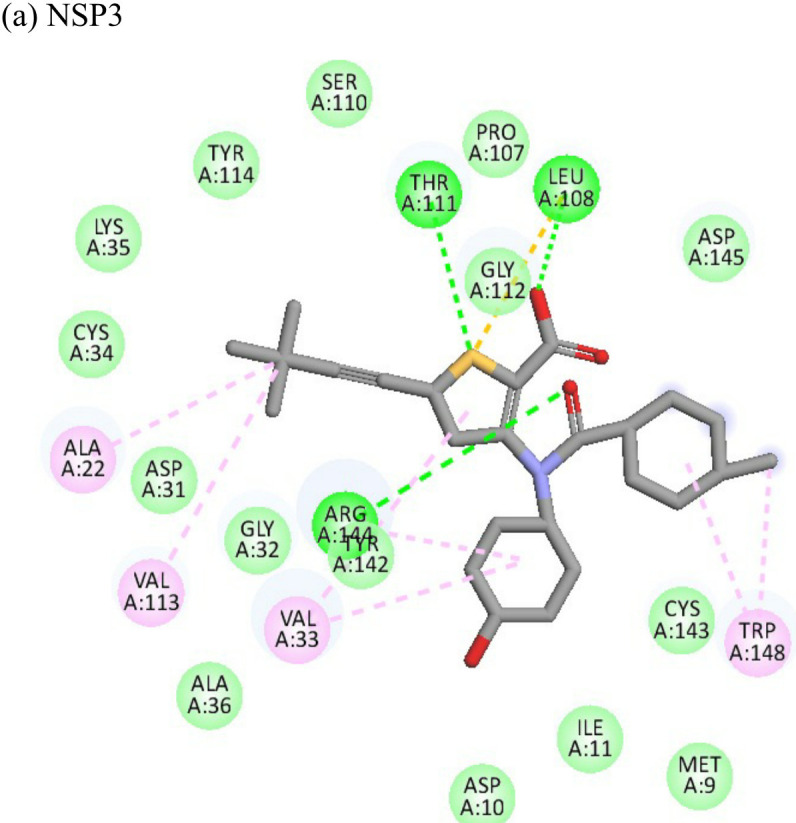	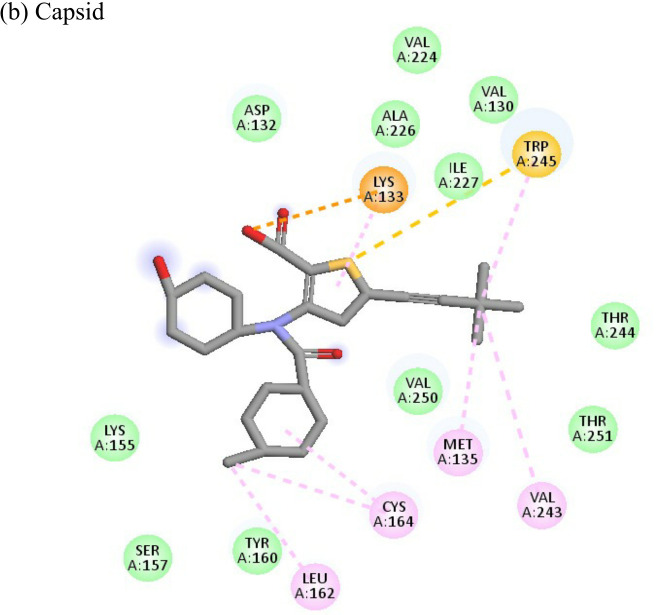	(a)-5.573 (b)-4.759	-81.13 -78.83	

The authors apologize for this error and state that this does not change the scientific conclusions of the article in any way. The original article has been updated.

