# Supplementary figures and images for: Drug repurposing approach against chikungunya virus: an in vitro and in silico study
Source: Front Cell Infect Microbiol. 2023 Apr 27;13:1132538. doi: 10.3389/fcimb.2023.1132538 (PMC10174255; doi:10.3389/fcimb.2023.1132538)

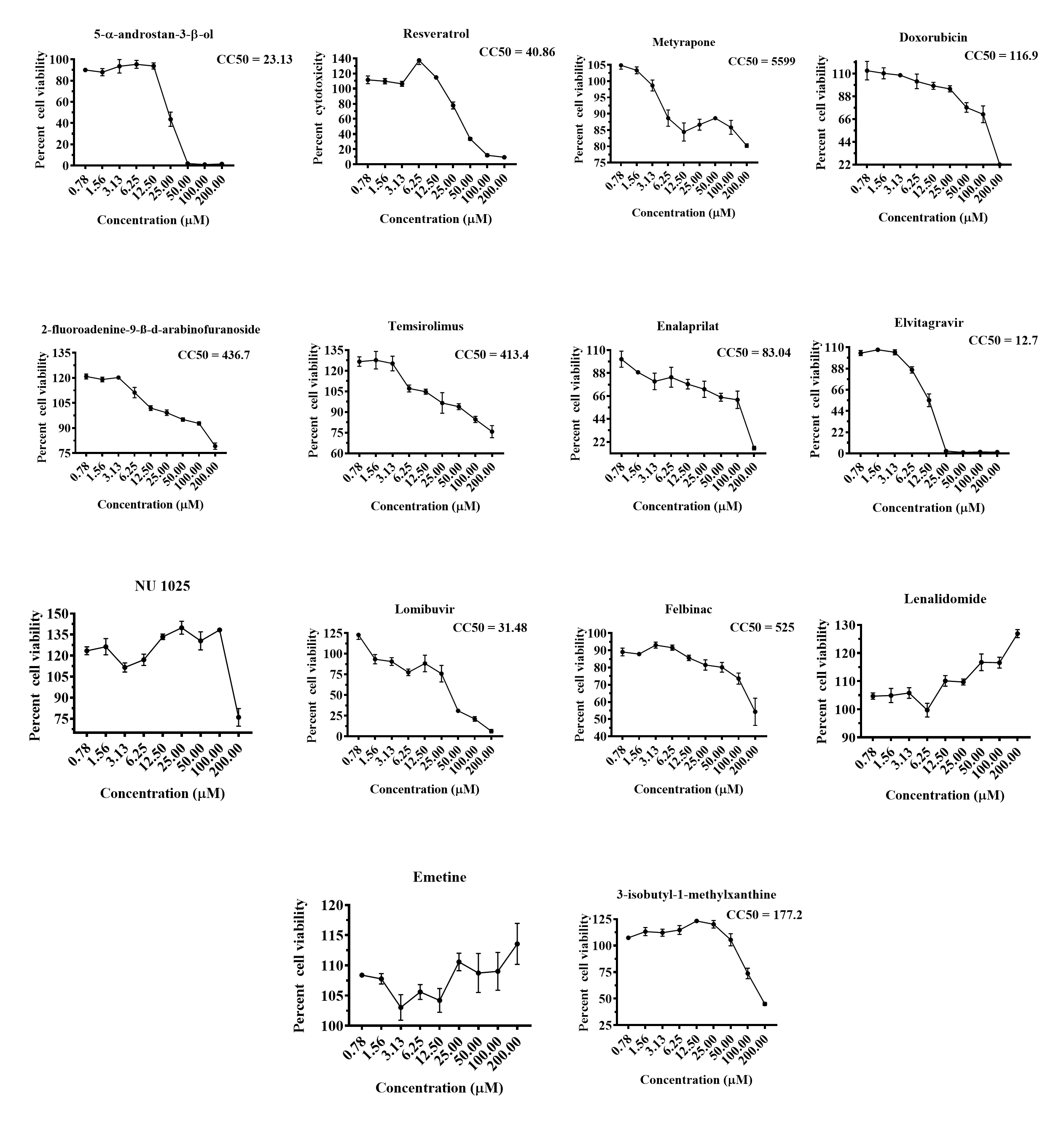

Supplement: Supplementary Figure 1 — Cytotoxic evaluation of selected drugs on Vero CCL-81 cells using MTT assay [file Image_1.tif]

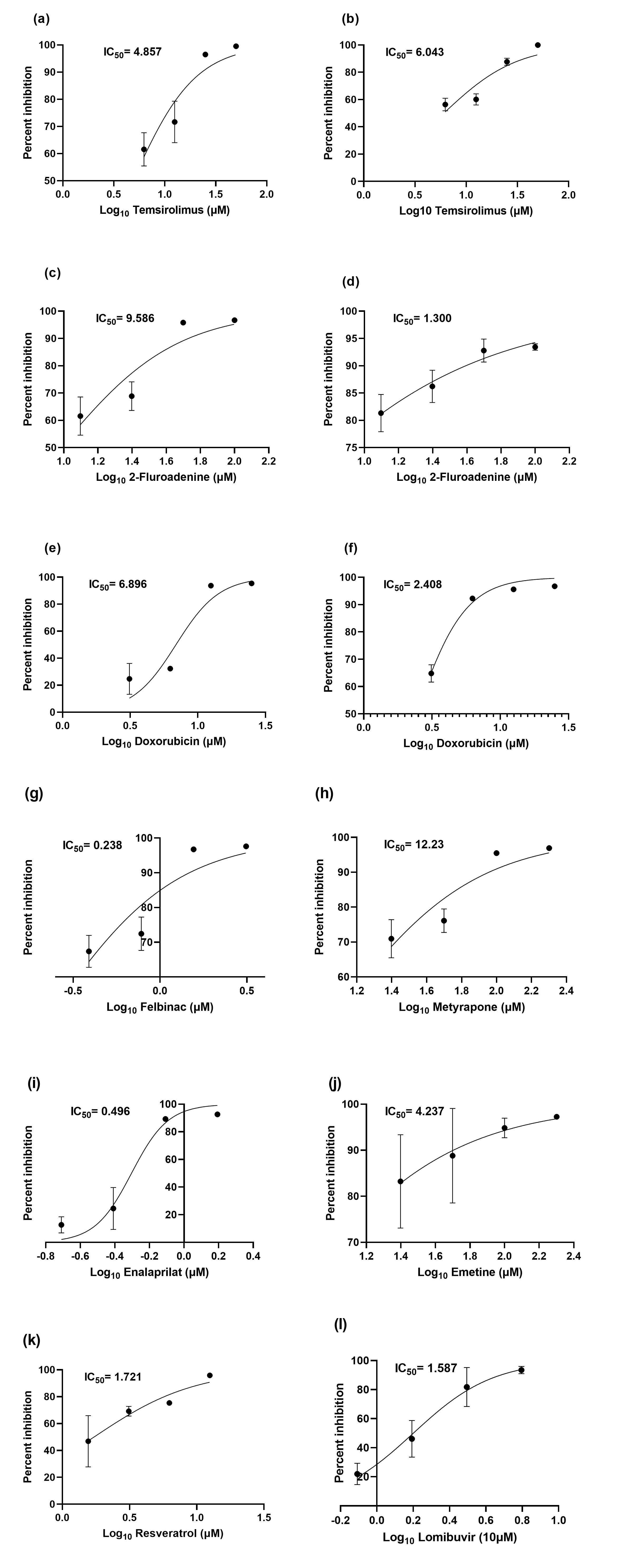

Supplement: Supplementary Figure 2 — Evaluation of percent inhibition and IC50 value. For finding out the IC50 value of the following drugs i.e. Temsirolimus (pretreatment) (A), Temsirolimus (posttreatment) (B), 2-fluoroadenine (pretreatment) (C), 2-fluoroadenine (posttreatment) (D), Doxorubicin (pretreatment) (E), Doxorubicin (posttreatment) (F), Felbinac (pretreatment) (G), Metyrapone (pretreatment) (H), Enalaprilat (cotreatment) (I), Emetine (posttreatment) (J), Resveratrol (K) (posttreatment), Lomibuvir (posttreatment) (L), percent inhibition of virus titre was calculated with reference to virus titre in VC and IC50 was calculated using non-linear regression analysis. All the values are represented as mean percent inhibition ± SE using VC as reference. ****p < 0.0001 vs VC. [file Image_2.tif]

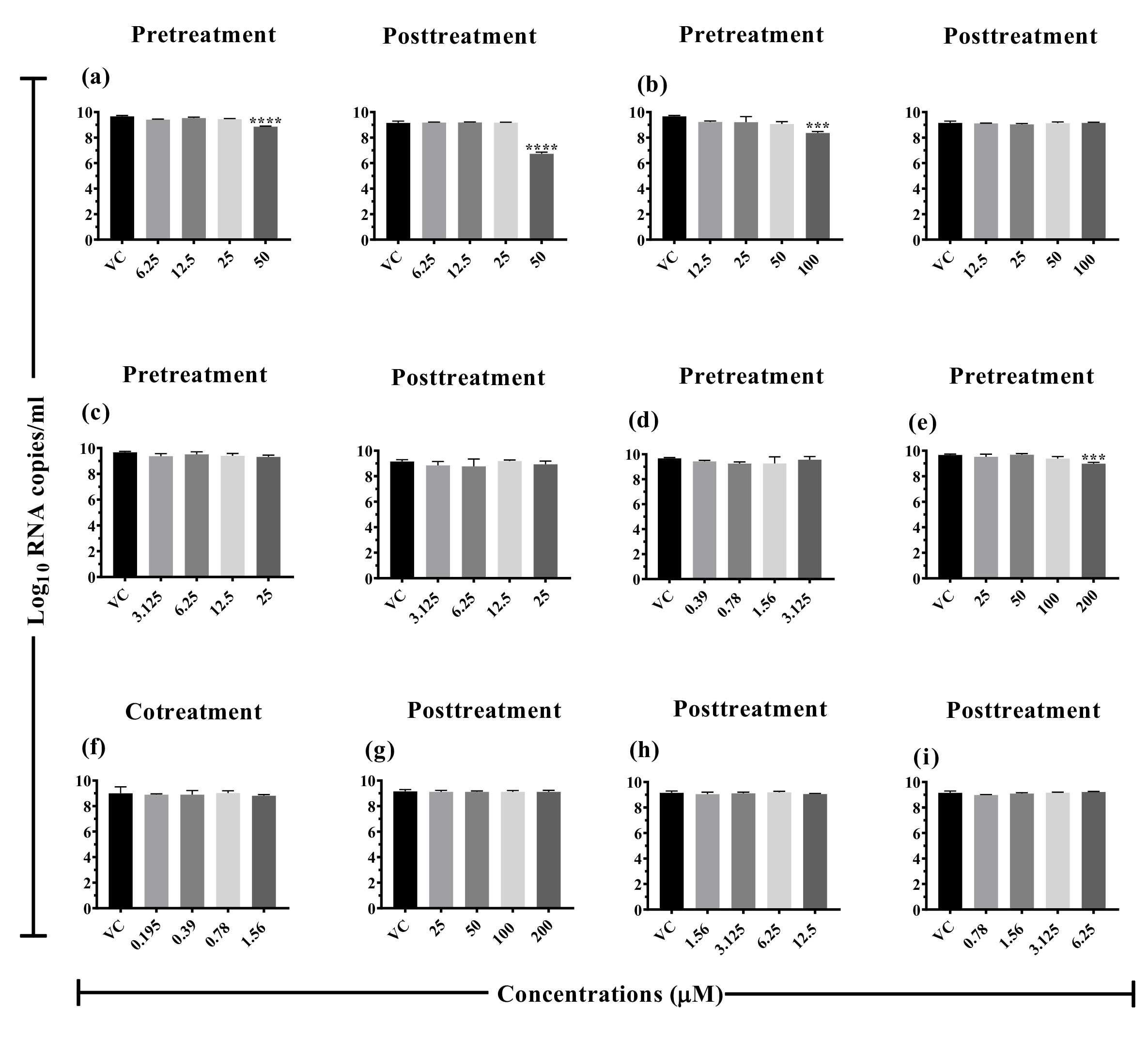

Supplement: Supplementary Figure 3 — Effect of different drugs on CHIKV RNA levels, quantified by qRT-PCR under different treatment conditions.Vero CCL–81 cells were pre, co and posttreated with different concentrations of drugs i.e. Temserolimus (A) 2-Fluroadenine (B) Doxorubicin (C) Felbinac (D) Metyrapone (E) Enalprilat (F) Ementine (G) Resveratrol (H) Lombubivir (I). After 24 h incubation, the plates were freezed and the culture filtrates were used for the qRT-PCR. Total RNA was isolated and CHIKV RNA was detected by measuring E3 RNA copies by real-time RT-PCR.Results were expressed as mean log10 viral RNA copies/ml ± SE and presented logarithmically. All the treatment groups were compared with a virus control (VC) group (without treatment). ****p < 0.0001, ***p < 0.001 vs. VC. [file Image_3.tiff]

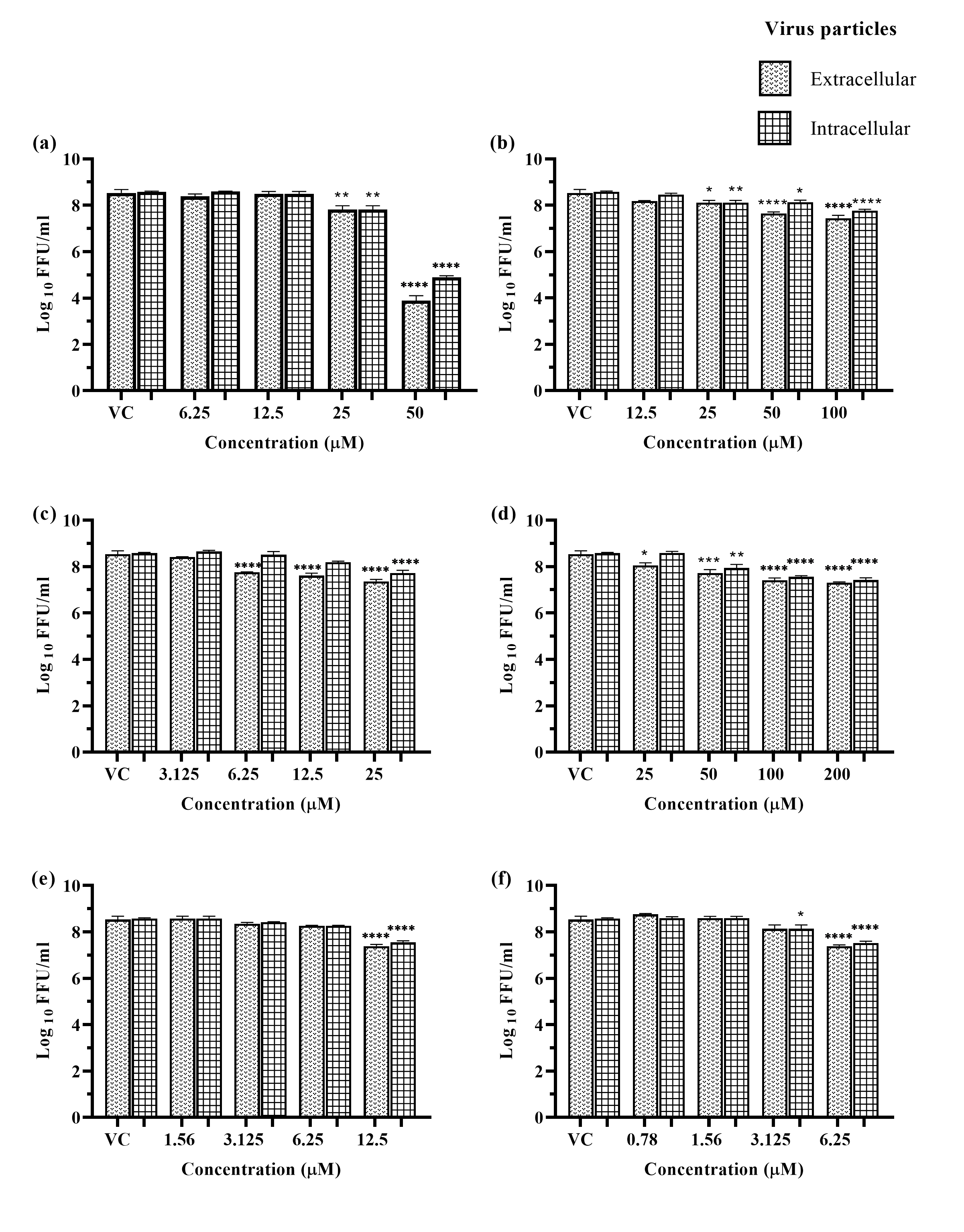

Supplement: Supplementary Figure 4 — Effect of different drugs added at 0 h time point post infection on the titer of extracellular and intracellular virus particles. Infected Vero CCL-81 cells were treated with different concentrations of drugs i.e. Temsorilmus (A), 2-Flouroadenine (B), Doxorubicin (C), Emetine (D), Resveratrol (E) and Lomibuvir (F) immediately post infection and incubated for 24 h post infection. Post incubation, culture supernatant and cells were separately assessed for virus titer using FFU assay. The outcomes are expressed as mean log10 foci forming unit/ml ± standard error. All the treatment conditions were compared with the VC. *p<0.05, **p<0.01, ***p<0.005, ****p<0.0001. [file Image_4.tif]

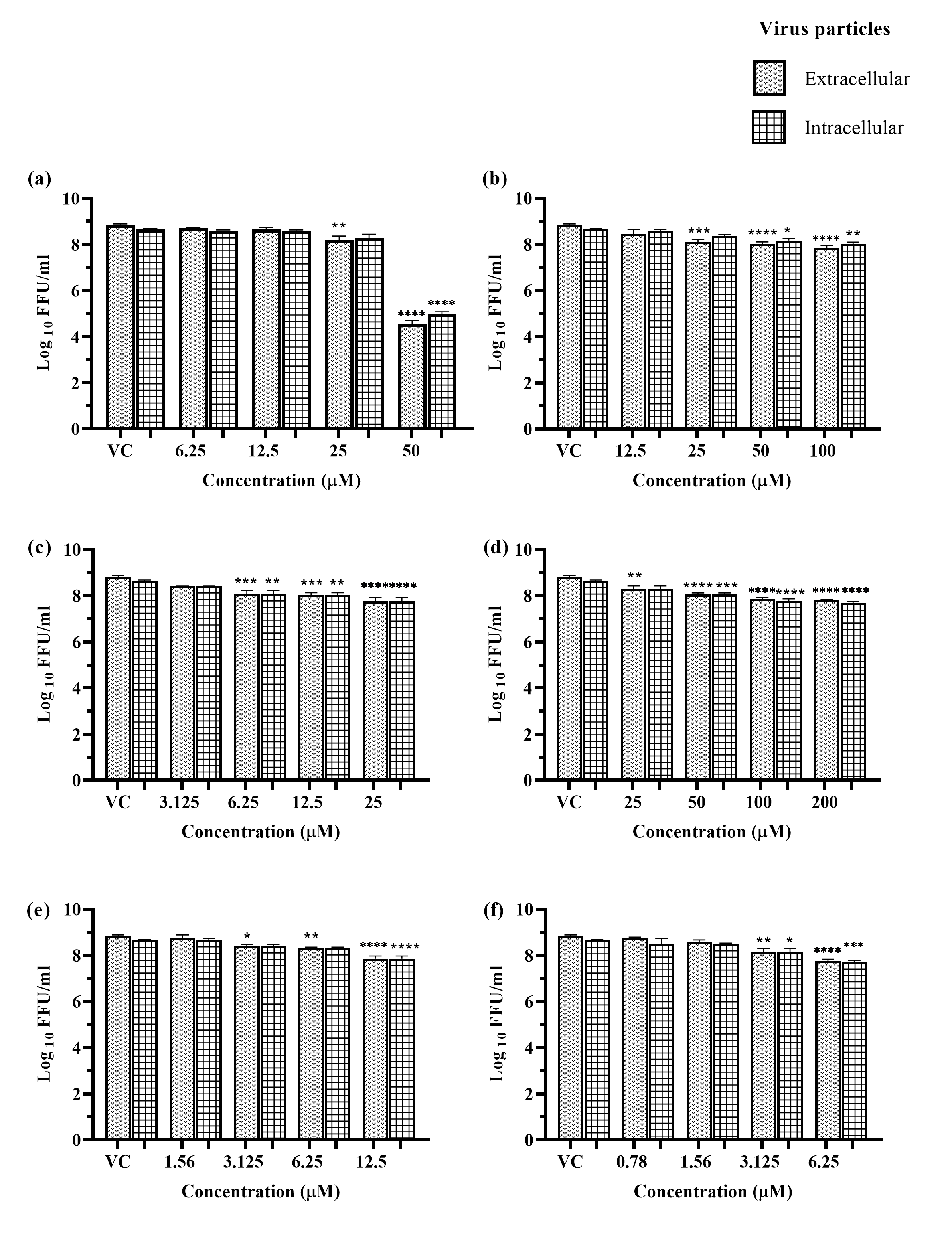

Supplement: Supplementary Figure 5 — Effect of different drugs added at 3 h time point post infection on the titer of extracellular and intracellular virus particles. Infected Vero CCL-81 cells were treated with different concentrations of drugs i.e. Temsorilmus (A), 2-Flouroadenine (B), Doxorubicin (C), Emetine (D), Resveratrol (E) and Lomibuvir (F) at 3h post infection and incubated for 24 h post infection. Post incubation, culture supernatant and cells were separately assessed for virus titer using FFU assay. The outcomes are expressed as mean log10 foci forming unit/ml ± standard error. All the treatment conditions were compared with the VC. *p<0.05, **p<0.01, ***p<0.005, ****p<0.0001. [file Image_5.tif]

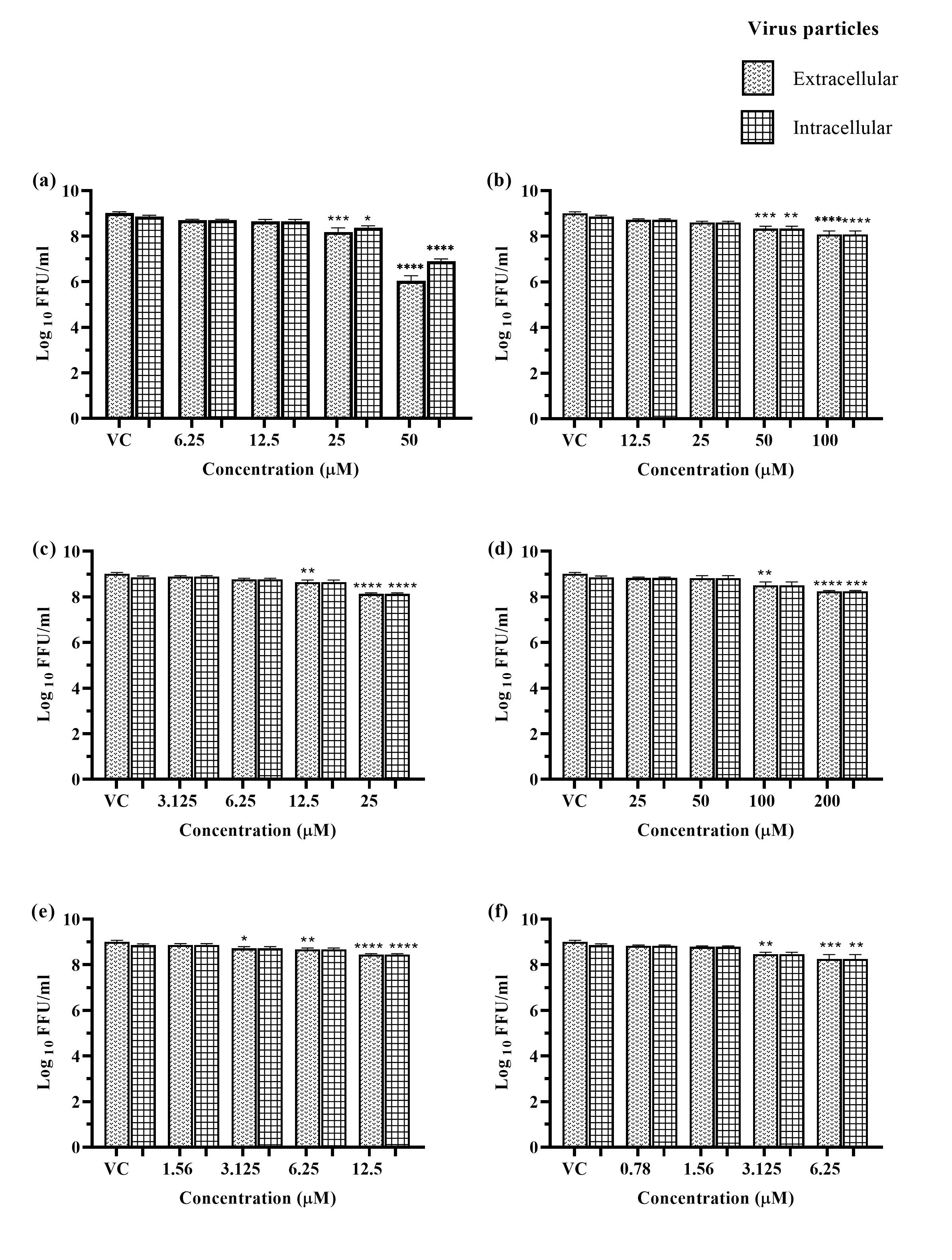

Supplement: Supplementary Figure 6 — Effect of different drugs added at 6 h time point post infection on the titer of extracellular and intracellular virus particles. Infected Vero CCL-81 cells were treated with different concentrations of drug i.e. Temsorilmus (A), 2-Flouroadenine (B), Doxorubicin (C), Emetine (D), Resveratrol (E) and Lomibuvir (F) at 6 h post infection and incubated for 24 h post infection. Post incubation, culture supernatant and cells were separately assessed for virus titre using FFU assay. The outcomes are expressed as mean log10 foci forming unit/ml ± standard error. All the treatment conditions were compared with the VC. *p<0.05, **p<0.01, ***p<0.005, ****p<0.0001. [file Image_6.tif]

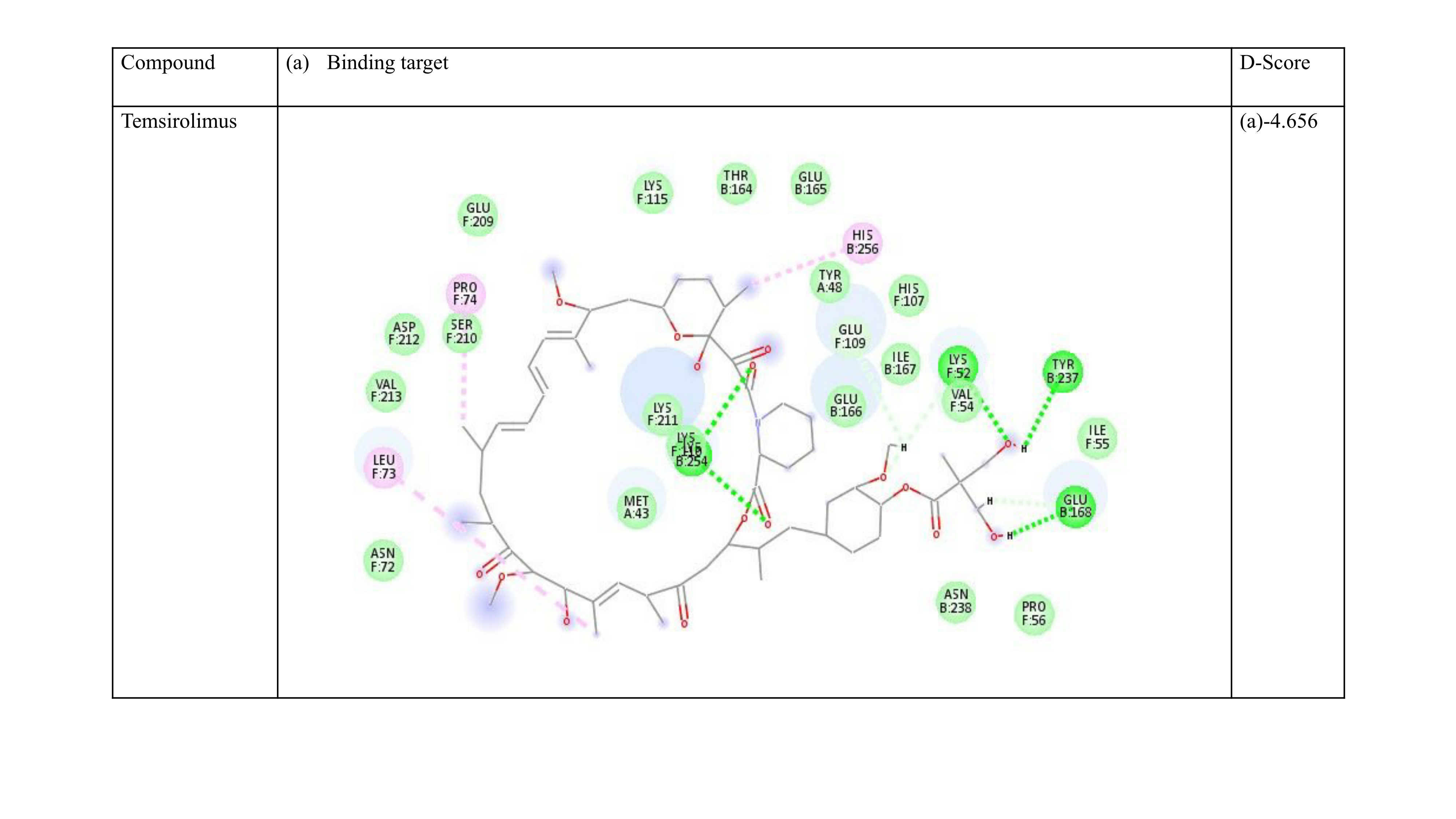

Supplement: Supplementary Figure 7 — Docking of temsirolimus to envelope protein and the resulting D-Score. [file Image_7.tif]
